# *Paecilomyces* and Its Importance in the Biological Control of Agricultural Pests and Diseases

**DOI:** 10.3390/plants9121746

**Published:** 2020-12-10

**Authors:** Alejandro Moreno-Gavíra, Victoria Huertas, Fernando Diánez, Brenda Sánchez-Montesinos, Mila Santos

**Affiliations:** Departamento de Agronomía, Escuela Superior de Ingeniería, Universidad de Almería, 04120 Almería, Spain; alejanmoga@gmail.com (A.M.-G.); victoriahuertas96@gmail.com (V.H.); fdianez@ual.es (F.D.); brensam@hotmail.com (B.S.-M.)

**Keywords:** biological control, diseases, pests, *Paecilomyces*

## Abstract

Incorporating beneficial microorganisms in crop production is the most promising strategy for maintaining agricultural productivity and reducing the use of inorganic fertilizers, herbicides, and pesticides. Numerous microorganisms have been described in the literature as biological control agents for pests and diseases, although some have not yet been commercialised due to their lack of viability or efficacy in different crops. *Paecilomyces* is a cosmopolitan fungus that is mainly known for its nematophagous capacity, but it has also been reported as an insect parasite and biological control agent of several fungi and phytopathogenic bacteria through different mechanisms of action. In addition, species of this genus have recently been described as biostimulants of plant growth and crop yield. This review includes all the information on the genus *Paecilomyces* as a biological control agent for pests and diseases. Its growth rate and high spore production rate in numerous substrates ensures the production of viable, affordable, and efficient commercial formulations for agricultural use.

## 1. Introduction

The genus *Paecilomyces* was first described in 1907 [1] as a genus closely related to *Penicillium* and comprising only one species, *P. variotii* Bainier. The description of this genus was revised by Brown and Smith [2], and Samson [3] defined 31 species divided into two sections: *Paecilomyces* characterized by thermophilic, thermotolerant, and mesophilic species, with yellow-brown colonies showing teleomorphic states corresponding to the genera *Byssochlamys*, *Talaromyces*, and *Thermoascus*; and *Isarioidea* characterized by mesophilic species with purple, pink, yellow, or green colonies. The former section includes the nematophagous or entomopathogenic species, also known as *Paecilomyces lilacinus* or *P. fumosoroseus* [4,5]. The different species in *Paecilomyces* are related to two genera of fungi: *Cordyceps* and *Torrubiella* [3].

Studies carried out by Luangsa-ard et al. [6] and Inglis and Tigano [4] confirm the polyphyletic origin of this genus that belongs to the Sordariomycetidae and Eurotiomycetidae subclasses. The Fungorum database [7] currently includes a list of 145 *Paecilomyces* species. Of all the species, some retain their original name, while others have been reclassified into other genera. One clear example is *Paecilomyces lilacinus* (Thom) Samson, which has been assigned to the genus *Purpureocillium* [8]. Despite its re-assignment to a different genus, *Paecilomyces lilacinus* will be included in this literature review, because of its importance in disease and pest control. Given the polyphyletic nature of the genus *Paecilomyces*, the evolution of these taxonomic studies is of great importance for developing microbial formulations that can be used in agriculture [9].

The genus *Paecilomyces* has hyaline to yellowish septate hyphae, often with smooth walls and verticillated or irregularly branched conidiophores, and phialides with a wide base and an elongated neck. The conidia are unicellular; hyaline, in chains; and the youngest conidium is at the basal end [10]. The conidial thermotolerance is correlated with their size and shape. Thus, the smaller and more spherical asexual conidia or ascospores are more vulnerable to high temperature [11,12,13,14]. *Paecilomyces* has high growth sporulation rates and grows over a wide range of temperatures and substrates. As a result, its rapid multiplication ensures viable and affordable development of commercial formulations [15].

The genus *Paecilomyces* has many species, both pathogenic and saprophytic, and can be found in a wide range of habitats, including soil [16,17], decomposing plant material or food [18,19], pasteurized food products [2,20,21], marine sediments [22,23], compost [24,25], insects [26,27,28,29], nematodes [30,31], or the rhizosphere of various plants [32,33], among others.

*Paecilomyces* also plays a significant role as an endophyte in numerous plants by providing several advantages for plant development. It can be used directly or indirectly as a potential biostimulant. When used directly, *Paecilomyces* or its metabolites increases the plant morphological parameters and crop yield [15,34,35,36,37]. The plant–*Paecilomyces* interaction improves plant health through different mechanisms and provides protection from phytopathogens [38]. This interaction showed a production of phytohormones, such as gibberellins and indole-acetic acid, that promoted growth and mitigated the effects of abiotic stress, such as salinity [39,40]. When used indirectly in combination with pathogenic agents such as nematodes or fungi, *Paecilomyces* has positive effects on crop growth by acting as a biological control agent [41,42,43,44].

**Figure 1:**
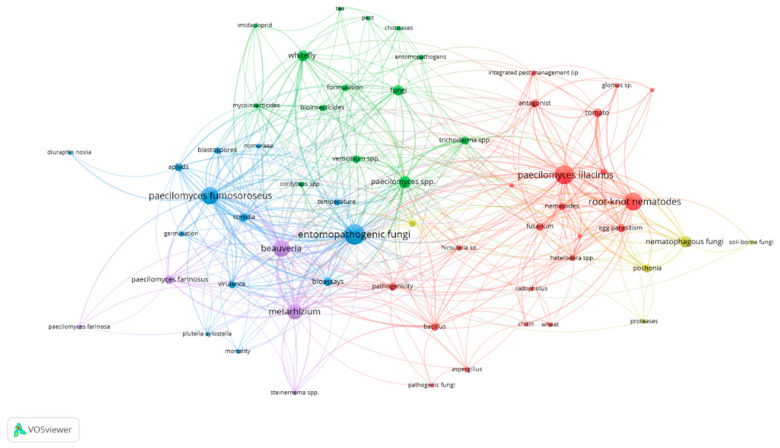
Network map of co-occurrence matrix for the 486 documents published in *Paecilomyces* research. VOSviewer software (version 1.6.15, Leiden University, Netherlands) was used to map the frequency of keyword co-occurrence networks. Differences in font size imply differences in relevance. The different colors refer to the groups or clusters formed.

Numerous species of the genus *Paecilomyces* produce a wide variety of secondary metabolites with different chemical structures and diverse biological activities, such as herbicidal [45], insecticidal [46,47], bactericidal [48], fungicidal [49], nematicidal [50,51,52] or cytotoxic [53]. There are also descriptions of metabolites with antitumour activity [54,55] or enzyme inhibitors, such as Paecilomide, which acts as an acetylcholinesterase inhibitor and can be used to control human diseases such as Alzheimer’s [56]. In addition, they have a role in aromatic compound degradation [57,58], ethanol production from agro-industrial wastes [59], or removal of ammonium from synthetic media or ammonia emission reduction in poultry manure [60,61]. Nevertheless, we cannot ignore the fact that *Paecilomyces* has been associated with several human infectious diseases in immunosuppressed patients [62,63] and has also been described as a phytopathogen. *P. variotii* was described by Aminaee et al. [64], as the causal agent of pistachio dieback, although subsequent molecular and phylogenetic studies reported that it was caused by *P. formosus* (Sakag., May., Inoue and Tada) Houbraken and Samsoninstead [65]. According to the map shown in Figure 1, there are few species of *Paecilomyces* responsible for the biological control of pests and diseases.

In this review, we will analyse the significant role of *Paecilomyces* in plant pest and disease control. In this sense, *Paecilomyces* is described as a biological control agent against bacteria, phytopathogenic fungi, nematodes, and numerous pests, using its extracts, secondary metabolites, or mycelium. To our knowledge, this is the first review of the genus *Paecilomyce*s as a biological control agent against plant pests and diseases.

## 2. Biological Control Mechanisms of the Genus *Paecilomyces*

Although many biological control mechanisms are unknown, advances in metagenomics provide some information on the plant–pathogen–antagonist interaction [66,67]. In the genus *Paecilomyces*, microbial mechanisms involved in pest and disease suppression have been direct, such as parasitism, competition or antibiosis, and indirect, which involve plant protection through induced systemic resistance (ISR) mechanisms [68,69,70].

### 2.1. Parasitism

*Paecilomyces* is capable of parasitizing fungi [71], nematodes and arthropods [72,73]. After recognition and pathogen-antagonist interaction take place, penetration and/or secretion of enzyme complexes occurs, leading to antagonist growth at the expense of its host [74,75]. Penetration can be mechanical, through appressoria development [76,77], or enzymatic, through cellulase, glucanase, laccase, leucinoxin, lipase, pectinase, protease, chitinase or xylanase release, which are involved in the infection process [78,79,80,81,82]. Thus, in vitro production of cellulases, lipases, and xylases by *P. tenuis* [83], chitinases and proteases by *P. fumosoroseus* (Wize) A.H.S. Br. and G. Sm. [84,85], or chitinolytic enzymes secreted by *P. lilacinus* [17] has been described. Chitinase production by *P. javanicus* leads to mycelia inhibition of *Aspergillus nidulans*, *Colletotrichum gloeosporioides*, *Rhizoctonia solani* and *Sclerotium rolfsii* [86].

On the other hand, Khan et al. [87] reported that lipases, proteases and chitinases have the strongest entomopathogenic effect. Thus, the production of these enzymes by *P. fumosoroseus* has been effective in the control of *Tenebrio molitor* [88], *Trialeurodes vaporariorum* [89] and *Plutella xylostella* [90]. *P. farinosus* (Holmsk.) A.H.S. Br. and G. Sm. proteases intervene in the control of *Galleria mellonella* [91].

Various studies refer to the nematicidal activity of *Paecilomyces.* Species of this genus, namely *P. lilacinus*, can penetrate both the eggshells and structural components of juvenile and adult stages of different species of nematodes through spore germination and subsequent hyphal branching and appresoria formation [92,93]. Regarding the production of lytic enzymes causing a nematicidal effect, the synthesis of amylases, lipases, proteases, and chitinases associated with this species has been described [77,78,85,87,94,95]. Overexpression of genes regulating the synthesis of these enzymes increases *P. lilacinus* virulence and parasitic ability against *Meloidogyne incognita, Panagrellus redivivus*, and *Caenorhabditis elegans* [96,97].

### 2.2. Competition

Competition for nutrients and space regulates the growth of pathogens coexisting in the same niche [67,82,98]. Siderophore production limits the availability of iron for pathogens [75,99]. In vitro synthesis of hydroxamate and carboxylate siderophores, such as ferrirubin trihydroxamate, has been described mainly in *P. lilacinus* and *P. variotii* [15,100,101,102,103,104].

While this mechanism has a direct impact on control, competition is often accompanied by other mechanisms [70]. The rapid growth of *Paecilomyces* species prevents the development of certain pathogens [105,106]. For instance, spraying sunflower seeds with *P. variotii* spores prevents penetration and infection by pathogen *Macrophomina phaseolina* [107]. However, this competition can sometimes have a negative impact on the rest of the beneficial microbiota [108].

### 2.3. Antibiosis

The production of secondary metabolites with antimicrobial effect by *Paecilomyces* species has been widely described. Among them, we can highlight the synthesis of alkaloids, phenolic compounds, volatile organic compounds, steroids, flavonoids, peptides, polyketides, quinones and terpenoids [109,110]. Li et al. [111] recently described a total of 148 active metabolites produced by different *Paecilomyces* species that can be used for drug or agrochemical development. In the following sections, we will show the importance of these metabolites in the biological control of pests and diseases.

### 2.4. Induced Resistance in Plants

The literature does not provide many examples on the effect of induced resistance after *Paecilomyces* colonizes the root system of a plant. Suárez-Estrella et al. [112] observed that inoculation of tomato plant roots with *P. variotii* significantly inhibited the signs caused by the bacterium *Xanthomonas campestris* on leaves. Similarly, López et al. [113] observed a reduction in the *Aphis gossypii* population in cotton plants whose seeds had been previously inoculated by being immersed in a *P. lilacinus* spore suspension. The combined use of *P. lilacinus* and salicylic acid improved the cellulose, hemicellulose, lignin, and pectin contents in cotton plants compared to inoculation treatments using *Pythium debaryanum* and *Fusarium oxysporum*, which showed that wall lignification provides a high level of protection against pathogen invasion. Likewise, concentration of soluble proteins and phenolic compounds increased in the root, which reduced the incidence of both diseases [114]. This also occurs when okra plants are inoculated with *P. lilacinus* [115].

Similarly, the effect of induced resistance can also be produced by *Paecilomyces* extracts. A commercial extract of *P. variotii* known as ZhiNengCong (ZNC) could also induce resistance against *Xanthomonas oryzae* or *Pseudomonas syringae* in rice plants or Arabidopsis, respectively. A dose of 500 ng/mL of ZNC could not inhibit the development of pathogens *in vitro*, while the use of a smaller dose of 100 ng/mL did generate immunity against said bacteria. On the other hand, reactive oxygen substances such as superoxide and hydrogen peroxide or callose also increase, compared to untreated Arabidopsis plants, in addition to activating salicylic acid synthesis, which is necessary for the defence response [36].

## 3. Biological Control of Diseases Caused by Phytopathogenic Bacteria

Few studies show the effectiveness of *Paecilomyces* against different species of phytopathogenic bacteria. *Paecilomyces variotii* isolated from municipal solid waste compost showed a reduction in 27% of diseases caused by *X. campestris* in melon, and a decrease in the pathogen population [112]. Nesha and Siddiqui [44] observed a reduction in soft rot and leaf blight caused by *P. carotovorum* pv. *carotovorum* and *X. campestris* pv. *carotae* after using *P. lilacinus*, alone or in combination with *A. niger* and an increase in the dry weight and chlorophyll content of a carrot crop.

Metabolites produced by this genus play a significant role in disease control due to its antagonistic effect, although there is little information on the matter compared to phytopathogenic bacteria. There are descriptions on the importance of antibacterial metabolites such as viriditoxin or betulin against non-phytopathogenic bacteria such as *S. aureus, Enterococcus sp. Micrococcus* sp., *Aeromonas Hydrophila, Flavobacterium sp, Pseudomonas aeruginosa*, and *Vibrio cholera* [116,117]. Sornakili et al. [83] recently reported the inhibition of *Erwinia carotovora, Xanthomonas oryzae* pv. *oryzae*, and *Ralstonia solanacearum* with in vitro inhibition between 13–45% using *P. tenuis*, an endophyte isolated from rice leaves. Various metabolites, such as octadecanoic acid, acetic acid, and 2-ethylhexyl ester, as well as enzymatic activities, xylanases, cellulases, and lipases, were involved in this control.

## 4. Biological Control of Diseases Caused by Phytopathogenic Fungi

**Figure 2:**
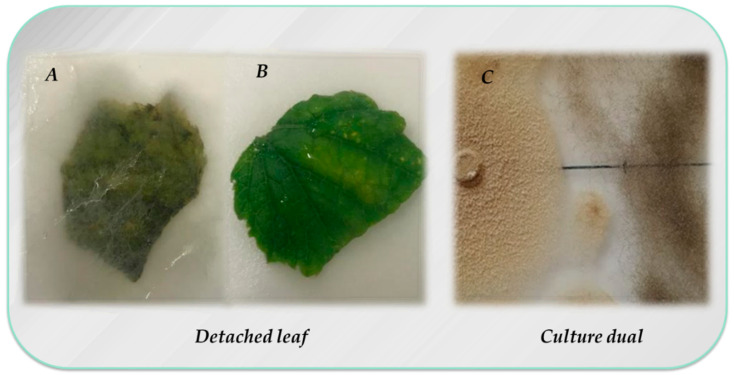
Detached leaf method to evaluate *P. variotii* as biological control agent against *B. cinerea*. (**A**) control leaves four days post infection with *B. cinerea*; (**B**) Leaf inoculated with spores *B. cinerea* and *P. variotii*. Photographs were taken four days after incubation in a moist petri dish at 20 °C under continuous white light. (**C**) Dual culture assay for in vitro inhibition of mycelial growth of *B. cinerea* by *P. variotii*.

Various *Paecilomyces* species have shown their antagonistic effect against phytopathogenic fungi causing root and aerial plant diseases through various mechanisms (Table 1). *P. variotii* and *P. lilacinus* species have proven to be quite effective, although most studies are *in vitro*. The antagonistic effect observed in most cases is explained by a competition for space and nutrients (Figure 2). However, other mechanisms associated with secondary metabolite production have been observed, which cause plasmolysis in spore germ tubes or hyphal melanisation in *Pyrenophora tritici-repentis* [118], hyphal lysis in *Moniliophthora roreri* caused by *Paecilomyces* sp. [71], mycoparasitism of *F. oxysporum* caused by *P. variotii* and *P. lilacinus* [119], or antibiosis against *R. solani* [120], among others. Viriditoxin, sphingofungins E and F [121], or eicosenoic acids are reported to have an antifungal effect against various phytopathogenic fungi such as *Biscogniauxia mediterranea, Phytophthora cinnamomi* or *Fusarium moniliforme* [61]. Varioxepin A or 6-Pentyl-α-pyrone inhibits perithecia formation and mycelial growth of *Fusarium graminearum* [122,123] or Paecylaminol, which inhibits soft rot development in tomatoes caused by *Mucor racemosus* [124].

Some in vivo studies show a direct effect on plant growth promotion after using *Paecilomyces*, [15] but also an indirect effect due to fungal disease control [72]. Yang et al. [125] observed inhibited *S. sclerotiorum* mycelial growth and sclerotia germination and a reduced disease severity after using *P. lilacinus* on a rapeseed crop. Results did not show differences after using spores or filtering without fungi cells, which highlighted the importance of *Paecilomyces* metabolites in pathogen control. In tomatoes, spraying *P. variotii* spores on the leaves significantly reduces damage caused by *Alternaria solani* [126]. On the other hand, the increase in polyphenols and antioxidant activity due to the use of *P. lilacinus* on okra roots improves plant development and control of various phytopathogenic fungi causing root rot [115]. Likewise, prior use of *P. fumosoroseus* delays the development of powdery mildew caused by *Podosphaera xanthii* [127], leading to mycelium and spore destruction due to the close contact of fungi and with some degree of mycoparasitism depending on the environmental conditions.

**Table 1:** Control of phytopathogenic fungi by *Paecilomyces* species.

Species	Phytopathogen	Assay/Plant	Reference
*Byssochlamys nivea*	*Rhizoctonia solani, Sclerotinia sclerotiorum,* *Aspergillus flavus*	In vitro	[128]
*P. farinosus*	*Blumeria graminis*	Dual culture, barley	[129]
	*Oidium neolycopersici*	Dual culture, tomato	
	*Golovinomyces orontii*	Dual culture, tobacco	
	*Podosphaera xanthii*	Dual culture, cucumber	
*P. fumosoroseus*	*Fusarium solani, R. solani, Sclerotium rolfsii* *Macrophomina phaseolina* *Pythium aphanidermatum*	Dual culture	[130] [43]
	*P. xanthii*	Cucumber	[127]
*P. lilacinus*	*R. solani*	Dual culture, poinsettia Sorghum, okra	[115,131]
		In vitro	[119]
	*Pyrenophora tritici-repentis*	Wheat	[118]
	*S. Sclerotiorum*	Dual culture, canola	[124]
	*A. flavus, A. parasiticus*	In vitro, soil	[132,133]
	*Magnaporthe oryzae*	Dual culture, rice	[134]
	*Fusarium oxysporum*	Chickpea Sorghum, okra	[135] [115]
	*S. sclerotiorum*	Wheat	[136]
	*F. oxysporum, P. debaryanum*	Cotton	[114]
	*R. bataticola*	Dual culture	[137]
	*F. chlamydosporum*	In vitro, tomato seeds	[42]
	*M. phaseolina, F. solani, F. oxysporum*	Dual culture, mung bean Okra	[115,138,139]
	*F. oxysporum f.sp. lycopersici*	Tomato	[72]
	*P. aphanidermatum,* *S. rolfsii*	In vitro	[43]
*P. marquandii*	*Verticilium dahliae*	Dual culture	[140]
	*R. solani*	Dual culture	[120]
*P. variotii*	*Pythium spinosum*	Dual culture, soybean	[141]
	*F. oxysporum*	Tomato	[142]
	*Biscogniauxia mediterránea, F. moniliforme, Phytophthora cinnamomi*	Rigid ryegrass	[61]
	*S. rolfsii, A.flavus*	Dual culture, in vitro	[43,131,143]
	*M. oryzae*	Dual culture	[133]
	*F. oxysporum*	Dual culture, chickpea	[134]
	*F. oxysporum*	Dual culture, melon	[112]
	*Alternaria solani, F. oxysporum*	Tomato	[126]
	*V. dahliae*	Dual culture	[106]
	*M. phaseolina*	Dual culture, sunflower	[107,138,144,145,146]
	*P. aphanidermatum*	Dual culture	[43]
	*F. oxysporum. f. sp. ciceris*	Chickpea	[134]
*Paecilomyces sp.*	*R. solani, S. sclerotiorum, A. flavus*	Dual culture	[126]
	*Moniliophthora roreri*	In vitro	[71]
	*Colletotrichum gloeosporoides*	Chili pepper	[146]
	*Phytophthora palmivora*	In vitro	[105]
	*F. graminearum*	In vitro	[122]
	*Ceratobasidium* *theobromae*	Cocoa	[147]
	*Mucor racemosus*	In vitro	[124]
*Paecilomyces spp.*	*Pyricularia oryzae*	In vitro	[28]
*P. sulphurellus*	*R. solani*	In vitro	[120]
*P. tenuis*	*M. phaseolina, M. grisea, Pythium sp., R. solani, F. oxysporum, Colletotrichum falcatum*	In vitro	[83]

## 5. Biological Control of Diseases Caused by Nematodes

As a nematophagus fungus, *Paecilomyces* has been widely studied and can be found in a variety of biological formulations for agricultural use [93]. There are many examples where *Paecilomyces* spp. act as nematicidal agents, especially against *Meloidogyne* spp., but also against other genera such as *Globodera* [52], *Rotylenchulus, Heterodera*, *Xiphinema* or *Pratylenchus* [51] (Table 2). One example is the use of *P. lilacinus* and *P. fumosoroseus* against *M. incognita* or *M. javanica*, which drastically reduces their populations [44,51,148,149], in both in vitro [43,87] and field tests [50,150]. The spores of these species must germinate on the host to penetrate and colonize its surface, in order to modify its physiology [51]. *Paecilomyces* acts according to the fungal and nematode species it parasitizes.

*Paecilomyces* spp. can act at different nematode developmental stages by infecting eggs, young or adult nematodes. Nematode eggshell is the main barrier against parasite agents and provides resistance to both chemical nematicides and biological compounds. *Paecilomyces* species are capable of secreting enzymes to degrade this barrier and deploying mechanisms involved in nematode parasitism [151,152]. Thus, observations have shown that *Meloidogyne incognita* eggs at early stages of development are more vulnerable than eggs containing fully developed juveniles, although the latter are also affected [153,154,155]. Hollan et al. [76] confirm that eggs are parasitized by *P. lilacinus* at all stages, including unhatched juveniles. Egg infection occurs when hyphae lie flat on the egg surface and appresoria are formed. Then, the fungus spreads and conidiophores are formed. Studies carried out by Khan et al. [92] concluded that said juveniles show various degrees of deformities and developmental abnormalities, such as reduced mobility inside the eggs. Different studies show the significant role of proteases and chitinases in the penetration of the fungus through eggshells. Thus, *M. arenaria* eggshells showed vitelline membrane disaggregation, and chitin and lipid layer destruction after using *P. lilacinus* [156].

Juvenile *M. hapla* eggs were highly vulnerable to serine proteases produced by *P. lilacinus* than eggs containing more developed juveniles. On the contrary, larvae showed no signs of damage. Jatala et al. [157] reported that *P.* *lilacinus* is capable of infecting female *Meloidogyne* spp. and *Heterodera* spp. and *Globodera* spp. cysts. In these cases, hyphae entered through natural openings of the body [158]. Evidence shows that various hydrolytic proteins, such as proteases (mainly serine proteases), collagenases and chitinases are involved in nematode cuticle penetration and subsequent cell degradation [77,97,159,160,161]. Likewise, different secondary metabolites produced by *Paecilomyces* also play a significant role in nematode control [162].

Nematode control effectiveness using *Paecilomyces* depends on the crop itself, as it affects fungal activity in many cases [163]. Thus, the use of an antagonist in combination with organic substances increases parasitism by *Paecilomyces* in both eggs and larvae of nematodes [164]. On the other hand, it has been reported that the use of *P. lilacinus* on recently solarised soil does not increase control effectiveness compared to non-solarised soil. However, a certain reduction in fungal activity is observed when both techniques are applied [165]. When comparing effectiveness using chemical compounds, *P. lilacinus* provides adequate control during crop growth, although the combination of both techniques shows better results compared to nematode control. [119,166,167,168,169].

As shown in Table 2, *P. lilacinus* is the most important nematophagous fungus, as it is capable of controlling various nematode species in different crops, though other species such as *P. marquandii* (Massee) S. Hughes [170,171,172] or *P. variotii* [173] can be equally effective. Reports by Chen et al. [171] on the use of *P. marquandii* against *M. hapla* showed an increase in lettuce weight, a decrease in gall formation by 25.7% and a reduction in egg production by 46.3%. According to Al-Assas, et al. [174], *P. variotii* reduces the number of galls by more than 90%, showing more effectiveness compared to chemical compounds.

**Table 2:** Nematode control by *Paecilomyces* species.

Species	Nematode	Assay/Plant	Reference
*P. fumosoreseus*	*Meloidogyne javanica*	In vitro	[51,175]
*P. lilacinus*	*M. enterolobii*	In vitro	[176]
	*M. arenaria*	Tomato	[177]
	*M. incognita*	Melon	[178]
		Tomato	[51,95,149,166,177,179,180,181,182,183,184,185,186,187,188]
		Eggplant	[181]
		Green beans	[163]
		Cotton, peanut, corn	[189,190]
		Cucumber	[44,191]
		In vitro	[95,154,155,162,192]
		Soybean	[193]
		Indian ginseng	[194]
		Carrot	[195]
		Potato	[153]
		Legumes	[196]
		In vitro	[197]
	*M. javanica*	Tomato	[168,169,198,199]
		Carrot	[44]
		In vitro	[87,175,200]
		Cherry	[201]
	*M. hapla*	In vitro, tomato	[192,202,203,204]
	*M. exigua*	Rubber tree	[205]
	*M. graminicola*	Wheat	[206]
	*M. marylandi*	Grass	[207]
	*M. paranaensis*	Coffee Tomato	[208] [209]
	*Meloidogyne spp.*	In vitro	[173,210,211]
		Tomato	[165]
	*Heterodera avenae*	In vitro, soil	[92,212,213]
	*H. glycines*	In vitro, Cotton Soybean, Wheat	[190,214] [215]
	*H. schachtii*	In vitro	[92,213]
	*H. trifolii*	Tomato	[216]
	*Heterodera spp.*	Potato	[157]
	*Globodera pallida*	In vitro	[119]
	*Globodera spp.*	Potato	[157]
	*Pratylenchus thornei*	In vitro, wheat	[157,217]
	*Pratylenchus spp.*	Sugar cane	[218]
		Cotton	[219]
	*Rotylenchulus reniformis*	Tomato	[152]
		In vitro, cotton	[210,220]
	*Tylenchulus semipenetrans*	In vitro	[221,222]
	*Radopholus similis*	Banana	[167]
	*R. reniformis*	In vitro	[210]
*P. marquandii*	*M. hapla*	Lettuce	[171]
	*M. hapla*	Lettuce	[172]
	*R. similis, H. multicinctus*	Banana	[223]
	*M. incognita*	Tomato	[170]
*P. variotii*	*Meloidogyne spp.*	In vitro	[173]
*Paecilomyces spp.*	*Meloidogyne spp.*	In vitro	[197]
	*G. rostochiensis*	Bean, chickpea	[224]
	*M. incognita*	Cucumber	[162]

## 6. Biological Control of Diseases Caused by Arthropods

The genus *Paecilomyces* includes multiple species described as pest control agents capable of providing natural control without the need for exogenous applications [225], many of which have been tested under controlled conditions for elaborating bioinsecticides to fight pests of great economic importance worldwide [226,227]. Among entomopathogenic fungi, *Paecilomyces* species are viable sources to elaborate mycoinsecticides, as affordable stable propagule substrates such as blastospores or conidia can be easily produced on a large scale [228]. According to Ruiu [229], bioformulations containing mainly *P. lilacinus* and *P. fumosoroseus* have been commercialised for pest control.

However, initial results obtained under in vitro culture conditions were not always consistent when assessing their effectiveness under field conditions. For this reason, parameters such as the application method should be assessed. In this case, most tests under controlled laboratory conditions are assessed by immersing samples in *Paecilomyces* spp. conidia suspensions, which provide clear results on the infectivity of the tested species [230]. Then, in planta tests are conducted under semi-natural conditions to assess effectiveness by spraying infected seedlings with conidial suspensions inside closed structures to prevent insects from going in or out [231]. Finally, mortality is assessed under field conditions by sprinkling crops showing a specific pest density with pre-commercial *Paecilomyces* spp. formulations [232]. In this sense, new application methods are currently being assessed, such as the one described by López et al. [113], where *P. lilacinus* used as an endophyte on cotton seeds provides induced resistance to plants by causing negative effects on *Aphis gossypii* feeding and reproduction.

Environmental conditions at the time of application are crucial and high temperatures and relative humidity are the most favourable for infection. In this sense, *P. fumosoroseus* caused a mortality of 60%, 80% and 85% in *Myzus persicae*, and of 90%, 95% and 100% in *Aphis fabae*, at 10 °C, 18 and 23 °C, respectively [233]. Regarding humidity, Demirci et al. [121] reported that *I. farinosa* showed increased pathogenicity against *Planococcus citri* under high relative humidity conditions at the time of application. Another aspect to bear in mind is the insects’ physiological state or size. Nymphal and larval stages tend to be more vulnerable than eggs as they have defence structures in their chorion. *P. fumosoroseus* is capable of affecting whitefly *Aleurodicus* *cocois* at various developmental stages [234]. Similarly, the physical barriers of *Leptinotarsa decemlineata* pupae make them more resistant than their larvae to *Isaria fumosorosea* infection [235]. In terms of size, Hunter et al. [236] showed a negative correlation between insect mortality and size mainly because larger sizes are associated with thicker cuticles, as in the use of *I. fumosorosea* on *Diaphorina citri* (psyllid) and *D. citri* (curculionidae), in which case the latter is bigger. Insect integument sclerotisation is also important, as it has an impact on *Paecilomyces* spp. ease of penetration and infection [237]. In order to avoid these obstacles, the use of formulations containing *Paecilomyces* spp. with high conidia densities is advised, as well as a focalised and prolonged exposure, to obtain an improved control effect against insects [238].

*Paecilomyces* has been described to control pests by limiting insect growth as a result of reduced feeding [236,239] reproduction [240] or simply causing their death due to mycosis [241]. In addition, it has been shown that *P. fumosoroseus* is capable of causing more deaths than some commercial insecticides such as fipronil when used against *Frankliniella occidentalis* [242]. Similar to when they act as nematophagous fungi, the potential of *Paecilomyces* spp. as a biological control agent that parasitises insects by penetrating their cuticle and subsequently spreading through haemolymph has been described [243]. This is possible owing to the excretion of enzymes, such as protease or chitinase synthesis [17,90], or different types of toxins, such as beauvericin [244], dipicolinic acid [46] or dibutyl succinate [245], which are described as bioactive metabolites with insecticidal or insect repellent effects, which turns them into significant virulence factors. Numerous orders of arthropods that are vulnerable to the use of *Paecilomyces* spp. (Table 3), including hemiptera, have been described, such as aleurodids [246], aphids [238], thysanoptera [242], diptera [247], lepidoptera [73], hymenoptera [248] and coleoptera [235].

## 7. Conclusions

The loss of pesticide effectiveness against certain pathogens, waste limitation in harvested products, the problems that these products cause to the environment and human health, and the ineffectiveness of genetic resistance due to quick alterations in pathogen virulence require the development of new control methods. While it is currently difficult to reduce the total amount of chemical active substances without causing losses in production, their gradual decrease and the use of bioestimulants can help optimize the use of chemical products and reduce environmental pollution. This review is the first to gather information on the potential of various *Paecilomyces* species as biological control agents against multiple diseases and pests, using different mechanisms of action and/or specificity that can be used in combination with cultural and chemical control in agriculture.

## Figures and Tables

**Table 3:** Biological control of pests using *Paecilomyces.*

Species	Pest	Assay/Plant	Reference
*P. carneus*	*Pteroma pendula*	In vitro	[249]
*P. cinnaomeus*	*Aleurocanthus camelliae*	In vitro	[250]
*P. javanicus, P. lilacinus*	*Spodoptera litura, Plutella xylostella*	In vitro	[73]
*P. farinosus*	*Sitophilus oryzae, Myzus persicae* *Lygus rugulipennis*	In vitro In vitro	[251,252] [253]
	*Planococcus citri*	In vitro	[254]
	*Tribolium confusum*	In vitro	[255]
	*Pristiphora abietina*	In vitro	[256]
	*Delia antiqua*	In vitro	[257]
	*Eurygaster integriceps*	Wheat	[258]
	*Hypothenemus* *hampei*	In vitro	[259]
	*Vespula, Dolichovespula*	Review	[260]
*P. formosa*	*Prays oleae*	In vitro	[261]
*P. fumosoroseus*	*Mamestra brassicae, S. littoralis*	In vitro	[262]
	*Hoplia philantus*	In vitro and grass	[263]
	*Monellia caryella, M. caryaefoliae* *M. pecanis*	In vitro	[264]
	*Diuraphis noxia*	In vitro	[265]
	*P. xylostella*	In vitro	[266]
	*Agriotes lineatus*	In vitro	[267]
	*Ceratitis capitata*	In vitro	[230,268]
	*Aphis fabae*	In vitro	[269]
	*Bemisia argentifolii*	Tomato, cabbage, cucumber	[270]
	*Diaphorina citri*	Orange, In vitro	[232,271]
	*Eutetranychus orientalis*	In vitro	[240]
	*Thrips palmi*	Bean	[272]
	*S. frugiperda*	Corn	[273]
	*Thrialurodes vaporariorum*	Tomato In vitro	[274] [275]
	*Bemisia tabaci*	In vitro	[276]
	*Tetranychus urticae*	Tomato	[277]
	*Toxoptera citricida*	In vitro	[278]
	*Hyalopterus pruni*	In vitro	[279]
	*Coccinelidos*	Review	[280]
	*Schizaphis* *graminum*	In vitro	[281]
	*B. tabaci*	Cotton	[282]
	*Anoplophora* *glabripennis*	In vitro	[283]
	*D. noxia*	Wheat	[284]
	*Delia radicum, D. floralis*	In vitro	[285]
	*Bactrocera zonata, B. cucurbitae*	In vitro	[286]
	*Haematobia irritans*	In vitro	[287,288]
	*Coptotermes curvignathus, C. gestroi*	In vitro	[241]
	*Leptinotarsa* *decemlineata*	In vitro	[235]
	*S. littoralis*	In vitro	[289]
	*Epilachna* *varivestis*	In vitro	[290]
	*Polyphagotarsonemus* *latus*	In vitro	[291]
	*B. argentifolii*	In vitro, hibiscus	[292,293,294,295,296]
	*P. xylostella*	In vitro	[297]
	*B. tabaci*	In vitro	[298]
	*B. tabaci, T. vaporariorum*	In vitro	[299]
	*Serangium* *parcesetosum*	In vitro	[300]
	*Drosophila suzukii*	In vitro	[301]
	*T. vaporariorum*	Tomato	[302]
*P. fumosoroseus* *P. lilacinus*	*Leptinotarsa* *decemlineata*	In vitro	[303]
*P. fumosoroseus* *P. farinosus*	*Rhagoletis* *cerasi*	In vitro	[304]
*P. fumosoroseus* *P. carneus* *P. lilacinus* *P. marquandii* *P. farinosus*	*Aedes aegypti*	In vitro	[305]
*P. lilacinus*	*Leptinotarsa* *decemlineata* *Phthorimaea* *operculella*	In vitro	[306]
	*Acromyrmex* *lundii*	In vitro	[248]
	*Aleurocanthus* *woglumi*	In vitro	[307]
	*Duponchelia* *fovealis*	In vitro	[308]
	*Rhipacephalus* *microplus*	In vitro	[309]
	*Tribolium* *confusum,* *Rhyzopertha* *dominica,* *Sitophilus* *zeamai*	In vitro	[29]
	*A. schlechtendali*	In vitro	[310]
	*T. vaporariorum, A. gossypii* *Frankliniella* *occidentalis* *Tetranychus* *urticae*	In vitro	[243]
	*Oligonychus* *coffeae*	In vitro	[311]
	*C. capitata*	In vitro	[312]
	*Galleria mellonella*	In vitro	[81,313]
	*A. gossypii*	Cotton	[113]
	*Solenopsis invicta*	In vitro	[314]
	*Tessaratoma* *papillosa*	In vitro	[315]
	*S. zeamais*	In vitro	[316]
	*Cyclocephala* *signaticollis*	In vitro	[317]
*P. lilacinus* *P. fumosoroseus*	*A. fabae*	In vitro	[318]
*P. niveus*	*Nasonovia* *ribisnigri*	In vitro	[319]
*P. tenuipes*	*S. frugiperda, S. exigua* *Helicoverpa* *zea, H. virescens*	In vitro	[320]
	*Otiorhynchus* *sulcatus*	In vitro	[321]
	*P. xylostela*	In vitro	[322]
*P. variotii*	*S. litura*	In vitro	[323]
	*S. avenae*	In vitro	[324]
	*Earias insulana*	In vitro	[325]
*Paecilomyces sp.*	*Lygus lineolaris*	In vitro	[326]
	*Carmenta foraseminis*	In vitro	[327]
	*Cyrtomenus* *bergi*	In vitro	[328]
	*Rhynchophorus* *ferrugineus*	In vitro	[329]
	*B. tabaci*	In vitro	[330]
	*S. litura*	In vitro	[331]
*Paecilomyces spp.*	*Hedypathes betulinus*	In vitro	[332]

## References

[1] Bainier G. (1907). Mycothe ‘que de l’e’cole de Pharmacie. XI *Paecilomyces*, genre nouveau de Muce’dine’es. Bull. Soc. Mycol. Fr..

[2] Brown A.H.S., Smith G. (1957). The genus *Paecilomyces* Bainier and its perfect stage *Byssochlamys* Westling. Trans. Br. Mycol. Soc..

[3] Samson R.A. (1974). *Paecilomyces* and some allied hyphomycetes. Stud. Mycol..

[4] Inglis P.W., Tigano M.S. (2006). Identification and taxonomy of some entomopathogenic *Paecilomyces* spp. (Ascomycota) isolates using rDNA-ITS Sequences. Genet. Mol. Biol..

[5] Ibarra J.E., Del Rincón C.M.C., Galindo E., Patiño M., Serrano L., García R., Carrillo J.A., Pereyra A.B., Alcázar P.A., Luna O.H. (2006). Los microorganismos en el control biológico de insectos y fitopatógenos. Rev. Latinoam. Microbiol..

[6] Luangsa-ard J.J., Hywel-Jones N.L., Samson R.A. (2004). The polyphyletic nature of *Paecilomyces* sensu lato based on 18S-generated rDNA phylogeny. Mycologia.

[7] Index Fungorum Database. http://www.indexfungorum.org/Names/Names.asp.

[8] Luangsa-Ard J.J., Houbraken J., Van Doorn T., Hong S.B., Borman A.M., Hywel-Jones N.L., Samson R.A. (2011). *Purpureocillium*, a new genus for the medically important *Paecilomyces lilacinus*. FEMS Microbiol. Lett..

[9] Obornik M., Jirku M., Dolezel D. (2001). Phylogeny of mitosporic entomopathogenic fungi: Is the genus *Paecilomyces* polyphyletic?. Can. J. Microbiol..

[10] Borba C.M., Brito M.M.S., Paterson R.R.M., Lima N. (2015). *Paecilomyces*: Mycotoxin production and human infection. Molecular Biology of Food and Water Borne Mycotoxigenic and Mycotic Fungi.

[11] Beuchat L.R. (1988). Influence of organic acids on heat resistance characteristics of *Talaromyces flavus* ascospores. Int. J. Food Microbiol..

[12] Dijksterhuis J. (2019). Fungal spores: Highly variable and stress-resistant vehicles for distribution and spoilage. Food Microbiol..

[13] Van den Brule T., Leeb C.L.S., Houbraken J., Haasc P.J., Wösten H., Dijksterhuis J. (2020). Conidial heat resistance of various strains of the food spoilage fungus *Paecilomyces variotii* correlates with mean spore size, spore shape and size distribution. Food Res. Int..

[14] Van den Brule T., Punt M., Teertstra W., Houbraken J., Wösten H., Dijksterhuis J. (2020). The most heat-resistant conidia observed to date are formed by distinct strains of *Paecilomyces variotii*. Environ. Microbiol..

[15] Moreno-Gavíra A., Diánez F., Sánchez-Montesinos B., Santos M. (2020). *Paecilomyces**variotii* as a plant-growth promoter in horticulture. Agronomy.

[16] He J., Kang J., Lei B., Wen T. (2011). *Paecilomyces wawuensis*, a new species isolated from soil in China. Mycotaxon.

[17] Homthong M., Kubera A., Srihuttagum M., Hongtrakul V. (2016). Isolation and characterization of chitinase from soil fungi, *Paecilomyces* sp. panel. Agric. Nat. Resour..

[18] M’barek H.N., Taidi B., Smaoui T., Aziz M.B., Mansouri A., Hajjaj H. (2019). Isolation screening and identification of ligno-cellulolytic fungi from northern central Morocco. Biotechnol. Agron. Soc. Environ..

[19] Biango-Daniels M.N., Snyderb A.B., Woroboc R.W., Hodge K.T. (2019). Fruit infected with *Paecilomyces niveus*: A source of spoilage inoculum and patulin in apple juice concentrate?. Food Control..

[20] Dagnas S., Membré J.M. (2013). Predicting and preventing mold spoilage of food products. J. Food Prot..

[21] Dos Santos J.L.P., Samapundo S., Biyikli A., Van Impe J., Akkermans S., Höfte M. (2018). Occurrence, distribution and contamination levels of heat-resistant moulds throughout the processing of pasteurized high-acid fruit products. Int. J. Food Microbiol..

[22] Wang Y., Zhang W.P., Cao H.L., Shek C.S., Tian R.M., Wong Y.H., Batang Z., Al-Suwailem A., Qian P.Y. (2014). Diversity and distribution of eukaryotic microbes in and around a brine pool adjacent to the Thuwal cold seeps in the Red Sea. Front. Microbiol..

[23] Rédou V., Navarri M., Meslet-Cladière L., Barbier G., Burgaud G. (2015). Species Richness and Adaptation of Marine Fungi from Deep-Subseafloor Sediments. Appl. Environ. Microbiol..

[24] Anastasi A., Varese G.C., Marchisio V.F. (2005). Isolation and identification of fungal communities in compost and vermicompost. Mycologia.

[25] Kluczek-Turpeinen B., Tuomela M., Hatakka A., Hofrichter M. (2003). Lignin degradation in a compost environment by the deuteromycete *Paecilomyces inflatus*. Appl. Microbiol. Biotechnol..

[26] Fukatsu T., Sato H., Kurivama H. (1997). Isolation, inoculation to insect host, and molecular phylogeny of an entomogenous fungus *Paecilomyces tenuipes*. J. Invertebr. Pathol..

[27] Marti G.A., López Lastra C.C., Pelizza S.A., García J.J. (2006). Isolation of *Paecilomyces lilacinus (*Thom) Samson (Ascomycota: Hypocreales) from the Chagas disease vector, *Triatoma infestans* Klug (Hemiptera: *Reduviidae*) in an endemic area in Argentina. Mycopathologia.

[28] Mohammadi S., Soltani J., Piri K. (2016). Soilborne and invertebrate pathogenic *Paecilomyces* species show activity against pathogenic fungi and bacteria. J. Crop Prot..

[29] Barra P., Rosso L., Nesci A., Etcheverry M. (2013). Isolation and identification of entomopathogenic fungi and their evaluation against *Tribolium confusum*, *Sitophilus zeamais*, and *Rhyzopertha dominica* in stored maize. J. Pest Sci..

[30] Aminuzzamana F.M., Xieb H.Y., Duanc W.J., Sundand B.D., Liu X.Z. (2013). Isolation of nematophagous fungi from eggs and females of *Meloidogyne* spp. and evaluation of their biological control potential. Biocontrol Sci. Technol..

[31] López-Lima D., Carrión G., Núñez-Sánchez A.E. (2014). Isolation of fungi associated with *Criconemoides* sp. and their potential use in the biological control of ectoparasitic and semiendoparasitic nematodes in sugar cane. Australian J. Crop Sci..

[32] Pandey A., Man L., Palni S., Bisht D. (2001). Dominant fungi in the rhizosphere of established tea bushes and their interaction with the dominant bacteria under in situ conditions. Microbiol. Res..

[33] Kilama P., Dubois T., Coyne D., Niere B., Gold C.S., Adipala E. (2007). Antagonism of *Paecilomyces* spp. isolated from banana (*Musa* spp.) roots and rhizosphere against *Radopholus similis*. Nematropica.

[34] Paul N.C., Deng J.X., Lee J.H., Yu S.H. (2013). New records of endophytic *Paecilomyces inflatus* and *Bionectria ochroleuca* from chili pepper plants in Korea. Mycobiology.

[35] Waqas M., Khan A.L., Shahzad R., Ullah I., Khan A.R., Lee I.J. (2015). Mutualistic fungal endophytes produce phytohormones and organic acids that promote japonica rice plant growth under prolonged heat stress. J. Zhejiang Univ Sci. B.

[36] Lu C., Liu H., Jiang D., Wang L., Jiang Y., Tang S., Hou X., Han X., Liu Z., Zhang M. (2019). *Paecilomyces variotii* extracts (ZNC) enhance plant immunity and promote plant growth. Plant Soil.

[37] Wang X., Yao Y., Chen B., Zhang M., Liu Z., Wang Q., Ma J. (2020). *Paecilomyces variotii* extracts and controlled-release urea synergistically increased nitrogen use efficiency and rice yield. ACS Omega.

[38] Malhadas C., Malheiro R., Pereira J.A., de Pinho P.G., Baptista P. (2017). Antimicrobial activity of endophytic fungi from olive tree leaves. World J. Microbiol. Biotechnol..

[39] Khan A.L., Hamayun M., Kang S.M., Kim Y.H., Jung H.Y., Lee J.H., Lee I.J. (2012). Endophytic fungal association via gibberellins and indole acetic acid can improve plant growth under abiotic stress: An example of *Paecilomyces formosus* LHL10. BMC Microbiol..

[40] Baron N.C., de Souza A., Rigobelo E.C. (2020). *Purpureocillium lilacinum* and *Metarhizium marquandii* as plant growth-promoting fungi. Peer J.

[41] Sivakumar T., Eswaran A., Balabaskar P. (2008). Bioefficacy of antagonists against for the management of *Fusarium oxysporum* f. sp*. lycopersici* and *Meloidogyne incognita* disease complex of tomato under field condition. Plant Arch..

[42] Mezeal I.A., Mizil S.N., Hussin M.S. (2018). Researching biocontrol of *Trichoderma viride*, *Paecilomyces lilacinus* in contradiction of effectiveness of fungi insulated as of selected therapeutic herbals. Plant Arch..

[43] Perveen Z., Shahzad S.A. (2013). Comparative study of the efficacy of *Paecilomyces* species against root-knot nematode *Meloidogyne incognita*. Pak. J. Nematol..

[44] Nesha R., Siddiqui Z.A. (2017). Effects of *Paecilomyces lilacinus* and *Aspergillus niger* alone and in combination on the growth, chlorophyll contents and soft rot disease complex of carrot. Sci. Hortic..

[45] Nakajima M., Itoi K., Takamatsu Y., Sato S., Furukawa Y., Furuya K., Honma T., Kadotani J., Ozasa M., Haneishi T. (1991). Cornexistin: A new fungal metabolite with herbicidal activity. J. Antibiot..

[46] Asaff A., Cerda-García-Rojas C., De la Torre M. (2005). Isolation of dipicolinic acid as an insecticidal toxin from *Paecilomyces fumosoroseus*. Appl. Microbiol. Biotechnol..

[47] Song X.B., Zhang L.H., Peng A.T., Cheng B.P., Ling J.F. (2016). First report of *Paecilomyces variotii* isolated from *Citrus Psyllid* (*Diaphorina citri*), the vector of Huanglongbing of Citrus, in China. Plant Dis..

[48] Hyung J.J., Kang H., Jong J.J., Soo K.Y. (2013). *Paecilomyces variotii* Extracts for Preventing and Treating Infections Caused by Fish Pathogenic Microorganisms. KR Patent.

[49] Pia̧tkowski J., Krzyzewska U., Nawrot U. (2003). Antifungal activity of enthomopathogenic species of the genus *Paecilomyces*. Mikol. Lek..

[50] Saha M., Sarkar S., Sarkar B., Sharma B.Q., Bhattacharjee S., Tribedi P. (2016). Microbial siderophores and their potential applications: A review. Environ. Sci. Pollut. Res..

[51] Favre-Bonvin J., Ponchet M., Djian C., Arpin N., Pijarowski L. (1991). Acetic acid: A selective nematicidal metabolite from culture filtrates of *Paecilomyces lilacinus* (Thom) Samson and *Trichoderma longibrachiatum* Rifai. Nematologica.

[52] Lima-Rivera D.L., Lopez-Lima D., Desgarennes D., Velazquez-Rodriguez A.S., Carrion G. (2016). Phosphate solubilization by fungi with nematicidal potential. J. Soil Sci. Plant Nutr..

[53] Kyong S.N., Young S.J., Yong H.K., Jin W.H., Ha W.K. (2001). Cytotoxic activities of ace-toxyscirpenediol and ergosterol peroxide from *Paecilomyces tenuipes*. Life Sci..

[54] Isaka M., Palasarn S., Lapanun S., Sriklung K. (2007). Paecilodepsipeptide A, an antimalarial and antitumor cyclohexadepsipeptide from the insect pathogenic fungus *Paecilomyces cinnamomeus* BCC 9616. J. Nat. Prod..

[55] He L., Shi W., Liu X., Zhao X., Zhang Z. (2018). Anticancer action and mechanism of ergosterol peroxide from *Paecilomyces cicadae* fermentation broth. Int. J. Mol. Sci..

[56] Paul A., Teles C., Takahashi J.A. (2013). Paecilomide, a new acetylcholinesterase inhibitor from *Paecilomyces lilacinus*. Microbiol. Res..

[57] García-Peña I., Hernández S., Auria R., Revah S. (2005). Correlation of biological activity and reactor performance in biofiltration of toluene with the fungus *Paecilomyces variotii* CBS115145. Appl. Environ. Microbiol..

[58] García-Peña I., Ortiz I., Hernández S., Revah S. (2008). Biofiltration of BTEX by the fungus *Paecilomyces variotii*. Int. Biodeterior Biodegrad..

[59] Zerva A., Savvides A.L., Katsifas E.A., Karagouni A.D., Hatzinikolaou D.G. (2014). Evaluation of *Paecilomyces variotii* potential in bioethanol production from lignocellulose through consolidated bioprocessing. Bioresour. Technol..

[60] Liu Z., Liu G., Cai H., Shi P., Chang W., Zhang S., Zheng A., Xie Q., Ma J. (2016). *Paecilomyces variotii*: A Fungus Capable of Removing Ammonia Nitrogen and inhibiting Ammonia Emission from Manure. PLoS ONE.

[61] Rodrigo S., Santamaria O.S., Halecker S., Lledó M.S. (2017). Antagonism between *Byssochlamys spectabilis* (anamorph *Paecilomyces variotii*) and plant pathogens: Involvement of the bioactive compounds produced by the endophyte. Ann. Appl. Biol..

[62] Steiner B., Aquino V.R., Paz A.A., da Rocha Silla L.M., Zavascki A., Goldani L.Z. (2013). *Paecilomyces variotii* as an emergent pathogenic agent of pneumonia. Case Rep. Infect. Dis..

[63] Pastor F.J., Guarro J. (2006). Clinical manifestations, treatment and outcome of *Paecilomyces lilacinus* infections. Clin. Microbiol. Infect..

[64] Aminaee M.M., Ershad D. (1989). Die-Back of Young Shoots of Pistachios in Kerman 9th Plant Protection Congress of Iran.

[65] Torabi A., Bonjar G.H.S., Abdolshahi R., Pournamdaric M., Saadound I., Barkae E.A. (2019). Biological control of *Paecilomyces formosus*, the causal agent of dieback and canker diseases of pistachio by two strains of *Streptomyces misionensis*. Biol. Control..

[66] O’Brien P.A. (2017). Biological control of plant diseases. Australas. Plant Pathol..

[67] Dukare A.S., Paul S., Nambi V.E., Gupta R.K., Singh R., Sharma K., Vishwakarma R.K. (2019). Exploitation of microbial antagonists for the control of postharvest diseases of fruits: A review. Crit. Rev. Food Sci. Nutr..

[68] Di Francesco A., Martini C., Mari M. (2016). Biological control of postharvest diseases by microbial antagonists: How many mechanisms of action?. Eur. J. Plant Pathol..

[69] Lugtenberg B., Rozen D.E., Kamilova F. (2017). Wars between microbes on roots and fruits. F1000Research.

[70] Latz M.A., Jensen B., Collinge D.B., Jørgensen H.J. (2018). Endophytic fungi as biocontrol agents: Elucidating mechanisms in disease suppression. Plant Ecol. Divers..

[71] Suárez L.Y., Rangel A.L. (2013). Isolation of microorganisms for biological control of *Moniliophthora roreri*. Acta Agron..

[72] Munawar M., Khan S.A., Javed N., Haq I.U., Gondal A.S. (2015). Bio-management of tomato wilt complex caused by *Meloidogyne incognita* and *Fusarium oxysporum* f.sp. *lycopersici*. Nematology.

[73] Nguyen H.C., Thi Van Anh T.R.A.N., Nguyen Q.L., Nguyen N.N., Nguyen M.K., Nguyen N.T.T. (2017). Newly Isolated *Paecilomyces lilacinus and Paecilomyces javanicus* as novel biocontrol agents for *Plutella xylostella* and *Spodoptera litura*. Not. Bot. Horti. Agrobo..

[74] Rabea E.I., Badawy M.E.T., Stevens C.V., Smagghe G., Steurbaut W. (2003). Chitosan as antimicrobial agent: Applications and mode of action. Biomacromolecules.

[75] Talibi I., Boubaker H., Boudyach E.H., Ait Ben Aoumar A. (2014). Alternative methods for the control of postharvest citrus diseases. J. Appl. Microbiol..

[76] Williams K., Khan A., Holland R. (1999). Infection of *Meloidogyne javanica* by *Paecilomyces lilacinus*. Nematology.

[77] Morton O., Hirsch P., Kerry B. (2004). Infection of plant-parasitic nematodes by nematophagous fungi–A review of the application of molecular biology to understand infection processes and to improve biological control. Nematology.

[78] Park J.O., Hargreaves J.R., McConville E.J., Stirling G.R., Ghisalberti E.L., Sivasithamparam K. (2004). Production of leucinostatins and nematicidal activity of Australian isolates of *Paecilomyces lilacinus* (Thom) Samson. Lett. Appl. Microbiol..

[79] Prabavathy D., Valli N.C. (2012). Screening for extracellular enzymes and production of cellulase by an endophytic *Aspergillus* sp, using cauliflower stalk as substrate. Int. J. Appl. Bioeng..

[80] Sunitha V.H., Nirmala Devi D., Srinivas C. (2013). Extracellular enzymatic activity of endophytic fungal strains isolated from medicinal plants. World J. Agric. Res..

[81] Ibrahim A.A., Mohamed H.F., El-Naggar S.E.M., Swelim M.A., Elkhawaga O.E. (2016). Isolation and selection of entomopathogenic fungi as biocontrol agent against the greater wax moth, *Galleria mellonella* L. (Lepidoptera: Pyralidae). Egypt. J. Biol. Pest Control.

[82] Spadaro D., Droby S. (2016). Development of biocontrol products for postharvest diseases of fruit: The importance of elucidating the mechanisms of action of yeast antagonists. Trends Food Sci. Tech..

[83] Sornakili A., Thankappan S., Sridharan A.P., Nithya P., Uthandi S. (2020). Antagonistic fungal endophytes and their metabolite-mediated interactions against phytopathogens in rice. Physiol. Mol. Plant Pathol..

[84] Castellanos-Moguel J., González-Barajas M., Mier T., del Rocío Reyes-Montes M., Aranda E., Toriello C. (2007). Virulence testing and extracellular subtilisin-like (Pr1) and trypsin-like (Pr2) activity during propagule production of *Paecilomyces fumosoroseus* isolates from whiteflies (Homoptera: *Aleyrodidae*). Rev. Iberoam. Micol..

[85] Gortari M.C., Galarza B.C., Cazau M.C., Hours R.A. (2008). Comparison of the biological properties of two strains of *Paecilomyces lilacinus* (Thom) Samson associated to their antagonistic effect onto *Toxocara canis* eggs. Malays. J. Microbiol..

[86] Chen C.C., Kumar H.A., Kumar S., Tzean S.S., Yeh K.W. (2007). Molecular cloning, characterization, and expression of a chitinase from the entomopathogenic fungus *Paecilomyces javanicus*. Curr. Microbiol..

[87] Khan A., Williams K.L., Nevalainen H.K. (2004). Effects of *Paecilomyces lilacinus* protease and chitinase on the eggshell structures and hatching of *Meloidogyne javanica* juveniles. Biol. Control..

[88] Gómez C., Amaya I., de la Cruz-Quiroz R., Rodríguez-Herrera R., Aguilar C.N. (2017). Tenebrio molitor biomass as inducer of lipases and proteases produced by *Paecilomyces fumosoroseus*. Mex. J. Biotechnol..

[89] Castellanos-Moguel J., Cruz-Camarillo R., Aranda E., Mier T., Toriello C. (2008). Relationship between protease and chitinase activity and the virulence of *Paecilomyces fumosoroseus* in *Trialeurodes vaporariorum* (Hemiptera: *Aleyrodidae*). Rev. Mex. Micol..

[90] Ali S., Huang Z., Ren S. (2010). Production of cuticle degrading enzymes by *Isaria fumosorosea* and their evaluation as a biocontrol agent against diamondback moth. J. Pest Sci..

[91] Lopez-Llorca L.V., Carbonell T., Gomez-Vidal S. (2002). Degradation of insect cuticle by *Paecilomyces farinosus* proteases. Mycol. Prog..

[92] Khan A., Williams K.L., Nevalainen H.K. (2006). Infection of plant-parasitic nematodes by *Paecilomyces lilacinus* and *Monacrosporium lysipagum*. BioControl.

[93] Dong L.Q., Yang J.K., Zhang K.Q. (2007). Cloning and phylogenetic analysis of the chitinase gene from the facultative pathogen *Paecilomyces lilacinus*. J. Appl. Microbiol..

[94] Giné A., Sorribas F.J. (2017). Effect of plant resistance and BioAct WG (*Purpureocillium lilacinum* strain 251) on *Meloidogyne incognita* in a tomato–cucumber rotation in a greenhouse. Pest Manag. Sci..

[95] Singh S., Pandey R.K., Goswami B.K. (2013). Bio-control activity of *Purpureocillium lilacinum* strains in managing root-knot disease of tomato caused by *Meloidogyne incognita*. Biocontrol Sci. Technol..

[96] Wang J., Liu F., Pan C. (2010). Enhancing the virulence of *Paecilomyces lilacinus* against *Meloidogyne incognita* eggs by overexpression of a serine protease. Biotechnol. Lett..

[97] Yang J., Zhao X., Liang L., Xia Z., Lei L., Niu X. (2011). Overexpression of a cuticle-degrading protease Ver112 increases the nematicidal activity of *Paecilomyces lilacinus*. Appl. Microbiol. Biotechnol..

[98] Rodriguez R.J., White Jr J.F., Arnold A.E., Redman A.R.A. (2009). Fungal endophytes: Diversity and functional roles. New Phytol..

[99] Saraf M., Pandya U., Thakkar A. (2014). Role of allelochemicals in plant growth promoting rhizobacteria for biocontrol of phytopathogens. Microbiol. Res..

[100] Vala A.K., Vaidya S.Y., Dube H.C. (2000). Siderophore production by facultative marine fungi. Indian J. Mar. Sci..

[101] Renshaw J.C., Robson G.D., Trinci A.P., Wiebe M.G., Livens F.R., Collison D., Taylor R.J. (2002). Fungal siderophores: Structures, functions and applications. Mycol. Res..

[102] Baakza A., Dave B.P., Dube H.C. (2004). Chemical nature, ligand denticity and quantification of fungal siderophores. Indian J. Exp. Boil..

[103] Daghino S., Martino E., Vurro E., Tomatis M., Girlanda M., Fubini B., Perotto S. (2008). Bioweathering of chrysotile by fungi isolated in ophiolitic sites. FEMS Microbiol. Lett..

[104] Ruanpanun P., Tangchitsomkid N., Hyde K.D., Lumyong S. (2010). Actinomycetes and fungi isolated from plant-parasitic nematode infested soils: Screening of the effective biocontrol potential, indole-3-acetic acid and siderophore production. World J. Microbiol. Biotechnol..

[105] Adebola M.O., Amadi J.E. (2010). Antagonistic activities of *Paecilomyces* and *Rhizopus* species against the cocoa black pod pathogen (*Phytophthora palmivora*). Afr. Sci..

[106] Arora K., Sharma S., Krishna S.B., Adam J.K., Kumar A. (2017). Non-edible Oil cakes as a novel substrate for DPA production and augmenting biocontrol activity of *Paecilomyces variotii*. Front. Microbiol..

[107] Anis M., Abbasi M.W., Zaki M.J. (2010). Bioefficacy of microbial antagonists against *Macrophomina phaseolina* on sunflower. Pak. J. Bot..

[108] Yu Z., Zhang Y., Luo W., Wang Y. (2015). Root colonization and effect of biocontrol fungus *Paecilomyces lilacinus* on composition of ammonia-oxidizing bacteria, ammonia-oxidizing archaea and fungal populations of tomato rhizosphere. Biol. Fertil. Soils.

[109] Mousa W.K., Raizada M.N. (2013). The diversity of anti-microbial secondary metabolites produced by fungal endophytes: An interdisciplinary perspective. Front. Microbiol..

[110] Lugtenberg B.J., Caradus J.R., Johnson L.J. (2016). Fungal endophytes for sustainable crop production. FEMS Microbiol. Ecol..

[111] Li X.Q., Xu K., Liu X.M., Zhang P. (2020). A systematic review on secondary metabolites of *Paecilomyces* species: Chemical diversity and biological activity. Planta Med..

[112] Suárez-Estrella F., Arcos-Nievas M.A., López M.J., Vargas-García M.C., Moreno J. (2013). Biological control of plant pathogens by microorganisms isolated from agro-industrial composts. Biol. Control..

[113] Lopez D.C., Zhu-Salzman K., Ek-Ramos M.J., Sword G.A. (2014). The entomopathogenic fungal endophytes *Purpureocillium lilacinum* (formerly *Paecilomyces lilacinus*) and *Beauveria bassiana* negatively affect cotton aphid reproduction under both greenhouse and field conditions. PLoS ONE.

[114] Abo-Elyousr K.A., Hashem M., Ali E.H. (2009). Integrated control of cotton root rot disease by mixing fungal biocontrol agents and resistance inducers. Crop Prot..

[115] Shafique H.A., Sultana V., Ara J., Ehteshamul-Haque S., Athar M. (2015). Role of antagonistic microorganisms and organic amendment in stimulating the defense system of okra against root rotting fungi. Pol. J. Microbiol..

[116] Oliveira Silva M.R., Kawai K., Hosoe T., Campos Takaki G.M., Buarque Gusmão N., Fukushima K., Méndez-Vilas A. (2013). Viriditoxin, an antibacterial substance produced by mangrove endophytic fungus *Paecilomyces variotii*. Microbial Pathogens and Strategies for Combating Them: Science, Technology and Education.

[117] Barakat K.M.I., Saleh M.E. (2016). Bioactive Betulin produced by marine *Paecilomyces* WE3-F. J. Appl. Pharm. Sci..

[118] Larran S., Simon M.R., Moreno M.V., Siurana M.S., Perelló A. (2016). Endophytes from wheat as biocontrol agents against tan spot disease. Biol. Control..

[119] Jacobs H., Gray S.N., Crump D.H. (2003). Interactions between nematophagous fungi and consequences for their potential as biological agents for the control of potato cyst nematodes. Mycol. Res..

[120] Horn W.S., Smith J.L., Bills G.F., Raghoobar S.L., Helms G.L., Kurtz M.B. (1992). Sphingofungins E and F: Novel serinepalmitoyl transferase inhibitors from *Paecilomyces variotii*. J. Antibiot..

[121] Demirci E., Dane E., Eken C. (2011). In vitro antagonistic activity of fungi isolated from sclerotia on potato tubers against *Rhizoctonia solani*. Turk. J. Biol..

[122] El-Hasan A., Schöne J., Höglinger B., Walker F., Voegele R.T. (2018). Assessment of the antifungal activity of selected biocontrol agents and their secondary metabolites against *Fusarium graminearum*. Eur. J. Plant Pathol..

[123] Zhang P., Li X.M., Wang J.N., Wang B.G. (2015). Oxepine-containing diketopiperazine alkaloids from the algal-derived endophytic fungus *Paecilomyces variotii* EN-291. Helv. Chim. Acta.

[124] Ui H., Shiomi K., Suzuki H., Hatano H., Morimoto H., Yamaguchi Y., Masuma R., Sakamoto K., Kita K., Miyoshi H. (2006). Paecilaminol, a new nadh-fumarate reductase inhibitor produced by *Paecilomyces* sp. FKI-0550. J. Antibiot..

[125] Yang F., Abdelnabby H., Xiao Y. (2015). A mutant of the nematophagous fungus *Paecilomyces lilacinus* (Thom) is a novel biocontrol agent for *Sclerotinia sclerotiorum*. Microb. Pathog..

[126] Varma P.K., Gandhi S.K., Surender S. (2008). Biological control of *Alternaria solani*, the causal agent of early blight of tomato. J. Biol. Control..

[127] Kavková M., Čurn V. (2005). *Paecilomyces fumosoroseus* (Deuteromycotina: Hyphomycetes) as a potential mycoparasite on *Sphaerotheca fuliginea* (Ascomycotina: Erysiphales). Mycopathologia.

[128] Dolatabad H.K., Javan-Nikkhah M., Shier W.T. (2017). Evaluation of antifungal, phosphate solubilisation, and siderophore and chitinase release activities of endophytic fungi from *Pistacia vera*. Mycol. Prog..

[129] Szentiványi O., Varga K., Wyand R., Slatter H., Kiss L. (2006). *Paecilomyces farinosus* destroys powdery mildew colonies in detached leaf cultures but not on whole plants. Eur. J. Plant Pathol..

[130] Ramzan N., Noreen N., Shahzad S. (2014). Inhibition of in vitro growth of soil-borne pathogens by compost-inhabiting indigenous bacteria and fungi. Pak. J. Bot..

[131] Cartwright D.K., Benson D.M. (1995). Biological control of *Rhizoctonia* stem rot of poinsettia in polyfoam rooting cubes with *Pseudomonas cepacia* and *Paecilomyces lilacinus*. Biol. Control..

[132] Will M.E., Wilson D.M., Wicklow D.T. (1994). Evaluation of *Paecilomyces lilacinus*, chitin, and cellulose amendments in the biological control of *Aspergillus flavus* fungi. Biol. Fertil. Soils.

[133] Gupta S.C., Leathers T.D., Wicklow D.T. (1993). Hydrolyticenzymes secreted by *Paecilomyces lilacinus* cultured on sclerotia of *Aspergillus flavus*. Appl. Microbiol. Biotechnol..

[134] Hajano J.U.D., Lodhi A.M., Pathan M.A., Khanzada M.A., Shah G.S. (2012). In-vitro evaluation of fungicides, plant extracts and bio-controlagents against rice blast pathogen *Magnaporthe Oryzae* couch. Pak. J. Bot..

[135] Maitlo S.A., Rajput N.A., Syed R.N., Khanzada M.A., Rajput A.Q., Lodhi A.M. (2019). Microbial control of *Fusarium wilt* of chickpea caused by *Fusarium oxysporum* f. sp.*ciceris*. Pak. J. Bot..

[136] Singh D. (1991). Biocontrol of *Sclerotinia sclerotiorum* (Lib.) de Bary by *Trichoderma harzianum*. Trop. Pest Manag..

[137] Khan M.A., Khan S.A., Khan R.W. (2017). Root Rot Disease Complex of Cotton: A Menace to Crop in Southern Punjab and its Mitigation through Antagonistic Fungi. Pak. J. Zool..

[138] Mansoor F., Sultana V., Ehteshamul-Haque S. (2007). Enhancement of biocontrol potential of *Pseudomonas aeruginosa* and *Paecilomyces lilacinus* against root rot of mungbean by a medicinal plant *Launaea nudicaulis*. Pak. J. Bot..

[139] Shahzad S., Ghaffar A. (1989). Use of *Paecilomyces lilacinus* in the control of root rot and root- knot disease complex of okra and mungbean. Pak. J. Nematol..

[140] Berg G., Zachow C., Lottmann J., Götz M., Costa R., Smalla K. (2005). Impact of plant species and site on rhizosphere-associated fungi antagonistic to *Verticillium dahliae* Kleb. Appl. Environ. Microbiol..

[141] Al-sheikh H., Abdelzaher H.M.A. (2010). Isolation of *Aspergillus sulphureus*, *Penicillium islandicum* and *Paecilomyces variotii* from agricultural soil and their biological activity against *Pythium spinosum*, the Damping-Off Organism of Soybean. J. Biol. Sci..

[142] Abdul-Wahid O.A., Moustafa A.F., Ibrahim M. (2001). Integrated control of tomato *Fusarium*-wilt through implementation of soil solarization and filamentous fungi. J. Plant Dis. Prot..

[143] Aziz N.H., Shahin A.A.M. (1997). Influence of other fungi on aflatoxin production by *Aspergillus flavus* in maize kernels. J. Food Saf..

[144] Shahzad S., Ghaffar A. (1987). Field application of *Paecilomyces lilacinus* and furadan for the control of rootknot disease of okra and mung. Int. Nematol. Network Newsl..

[145] Qureshi S.A., Ruqqia V., Ara S.J., Ehteshamul-Haque S. (2012). Nematicidal potential of culture filtrates of soil fungi associated with rhizosphere and rhizoplane of cultivated and wild plants. Pak. J. Bot..

[146] Nurbailis N., Martinius M., Azniza V. (2018). Viability and environmental effect to conidial germination of antagonistic fungi that potential as biological control of *Colletotrichum gloeosporoides* caused antracnose disease on chili. Biodiversitas.

[147] Taufik M., Yusuf D.N., Boer D., Botek M. (2019). Evaluating the ability of endophyte fungus to control VSD diseases in cocoa seeding. IOP Conference Series: Earth and Environmental Science.

[148] Walters S.A., Barker K.R. (1994). Efficacy of *Paecilomyces lilacinus* in suppressing *Rotylenchulus reniformis* on tomato. J. Nematol..

[149] Siddiqui Z.A., Akhtar M.S. (2009). Effects of antagonistic fungi and plant growth promoting rhizobacteria on growth of tomato and reproduction of the root-knot nematode, *Meloidogyne incognita*. Australas. Plant Pathol..

[150] Brand D., Roussos S., Pandey A., Zilioli P.C., Pohl J., Soccol C.R. (2004). Development of a bionematicide with *Paecilomyces lilacinus* to control *Meloidogyne incognita*. Appl. Biochem. Biotechnol..

[151] Roumpos C. (2005). Ecological Studies on Paecilomyces Lilacinus Strain 251 and Their Importance for Biocontrol of Plant-Parasitic Nematodes and Environmental Risk Assessment.

[152] Sexton A.C., Howlett B.J. (2006). Parallels in fungal pathogenesis on plant and animal hosts. Eukaryot. Cell.

[153] Jatala P., Kaltenback R., Bocangel M., Devaus A.J., Campos R. (1980). Field application of *Paecilomyces lilacinus* for controlling *Meloidogyne incognita* on potatoes. J. Nematol..

[154] Eapen S.J., Beena B., Ramana K. (2005). Tropical soil microflora of spice-based cropping systems as potential antagonists of root-knot nematodes. J. Invertebr. Pathol..

[155] Dunn M.T., Sayre R.M., Carrell A., Wergin W.P. (1982). Colonization of nematode eggs by *Paecilomyces lilacinus* (Thom) Samson as observed with scanning electron microscope. Scan. Electron Microsc..

[156] Morgan-Jones G., White J.F., Rodriguez-Kabana R. (1984). Phyto-nematode pathology: Ultrastructural studies II. Parasitism of *Meloidogyne arenaria* eggs and larvae by *Paecilomyces lilacinus*. Nematropica.

[157] Jatala P., Kaltenback R., Bocangel M. (1979). Biological control of *Meloidogyne incognita* acrita and *Globodera pallida* on potatoes. J. Nematol..

[158] Jatala P. (1986). Biological controlof plant-parasitic nematodes. Ann. Rev. Phytopathol..

[159] Huang X.W., Zhao N.H., Zhang K.Q. (2004). Extracellular enzymes serving as virulence factors in nematophagous fungi involved in infection of the host. Res. Microbiol..

[160] Ahman J., Johanson T., Olsson M., Punt P.J., Van den Hondel C.A.M.J.J., Tunlid A.S. (2002). Improving the pathogenicity of a nematodetrapping fungus by genetic engineering of a subtilisin with nematotoxic activity. Appl. Environ. Microbiol..

[161] Pau C.G., Leong S., Teck C., Wong S.K., Eng L., Jiwan M. (2012). Isolation of indigenous strains of *Paecilomyces lilacinus* with antagonistic activity against *Meloidogyne incognita*. Int. J. Agric. Biol..

[162] Yan X.N., Sikora R.A., Zheng J.W. (2011). Potential use of cucumber (*Cucumis sativus* L.) endophytic fungi as seed treatment agents against root-knot nematode *Meloidogyne incognita*. J. Zhejiang Univ. Sci. B.

[163] Al-Hazmi A.S., Dawabah A.A.M., Al-Nadhari S.N., Al-Yahya F.A. (2017). Comparative efficacy of different approaches to managing *Meloidogyne incognita* on green bean. Saudi J. Biol. Sci..

[164] Siddiqui Z.A., Futai K. (2009). Biocontrol of *Meloidogyne incognita* on tomato using antagonistic fungi, plant-growth-promoting rhizobacteria and cattle manure. Pest Manag. Sci..

[165] Anastasiadis I.A., Giannakou I.O., Prophetou-Athanasiadou D.A., Gowen S.R. (2008). The combined effect of the application of a biocontrol agent *Paecilomyces lilacinus*, with various practices for the control of root-knot nematodes. Crop Prot..

[166] Dahlin P., Eder R., Consoli E., Krauss J., Kiewnick S. (2019). Integrated control of *Meloidogyne incognita* in tomatoes using fluopyram and *Purpureocillium lilacinum* strain 251. Crop Prot..

[167] Mendoza A.R., Sikora R.A., Kiewnick S. (2007). Influence of *Paecilomyces lilacinus* strain 251 on the biological control of the burrowing nematode *Radopholus similis* in banana. Nematropica.

[168] Zaki F.A., Bhatti D.S. (1999). Effect of Castor (*Ricinus Communis*) and the biocontrol fungus *Paecilomyces lilacinus* on *Meloidogyne Javanica*. Nematologica.

[169] A-Raddad A.M. (1995). Interaction of *Glomus mosseae* and *Paecilomyces lilacinus* on *Meloidogyne javanica* of tomato. Mycorrhiza.

[170] Marban-Mendoza N., Garcia-E R., Dicklow M.B., Zuckerman B.M. (1992). Studies on *Paecilomyces marquandii* from nematode suppressive chinampa soils. J. Chem. Ecol..

[171] Chen J., Abawi G.S., Zuckerman B.M. (2000). Efficacy of *Bacillus thuringiensis*, *Paecilomyces marquandii*, and *Streptomyces costaricanus* with and without organic amendments against *Meloidogyne hapla* infecting lettuce. J. Nematol..

[172] Chen J., Abawi G.S., Zuckerman B.M. (1999). Suppression of *Meloidogyne hapla* and its damage to lettuce grown in a mineral soil amended with chitin and biocontrol organisms. J. Nematol..

[173] Ahmad R.Z., Sidi B.B., Endrawati D., Ekawasti F., Chaerani C. (2019). *Paecilomyces lilacinus* and *P variotii* as a predator of nematode and trematode eggs. IOP Conf. Ser. Earth. Environ. Sci..

[174] Al-Assas K.M.K., Naffaa W. (2011). Effectiveness of *Paecilomyces variotii*, Plant Extraction of Hemlock Conium maculatum and Some Pesticides in Controlling Root-Knot Nematode *Meloidogyne incognita* on Tomato. Arab. J. Arid. Environ..

[175] Tigano-Milano S., Carneiro G., De Faria R., Frazao S., Mccoy C. (1994). Isozyme Characterization and Pathogenicity of *Paecilomyces fumosoroseus* and *P. lilacinus* to *Diabrotica speciosa* (Coleoptera Chrysomelidae) and *Meloidogyne javanica* (Nematoda: Tylenchidae). Biol. Control..

[176] Carneiro G., Hidalgo-Díaz L., Martins I., Ayres De Souza silva K.F., Milano-Tigano S. (2011). Effect of nematophagous fungi on reproduction of *Meloidogyne enterolobii* on guava (*Psidium guajava*) plants. Nematology.

[177] Kepenekci I., Oksal E. (2015). Evaluation of entomopathogenic fungi, *Purpureocillium lilacinum* TR1 for the control of the Root-knot nematodes (*Meloidogyne javanica*, *M. incognita and M. arenaria*). Türk. Entomol. Derg..

[178] Abdeldaym E.A., Erriquens F., Verrastro V., Sasanelli N., Mondelli D., Cocozza C. (2012). Nematicidal and fertilizing effects of chicken manure, fresh and composted olive mill wastes on organic melon. Helminthologia.

[179] Kiewnick S., Sikora R.A. (2006). Evaluation of *Paecilomyces lilacinus* strain 251 for the biological control of the northern root-knot nematode *Meloidogyne hapla* Chitwood. Nematology.

[180] Liu J., Sun J., Qiu J., Liu X., Xiang M. (2014). Integrated management of root-knot nematodes on tomato in glasshouse production using nematicides and a biocontrol agent, and their effect on soil microbial communities. Nematology.

[181] Mittal N., Saxena G., Mukerji G.K. (1995). Integrated control of root-knot disease in three crop plants using chitin and *Paecilomyces lilacinus*. Crop Prot..

[182] Kiewnick S., Neumann S., Sikora R.A., Frey J.E. (2011). Importance of nematode inoculum density and antagonist dose for biocontrol efficacy of *Paecilomyces lilacinus* strain 251. Phytopathology.

[183] Siddiqui Z.A., Akhtar M.S. (2008). Synergistic effects of antagonistic fungi and a plant growth promoting rhizobacterium, an arbuscular mycorrhizal fungus, or composted cow manure on populations of *Meloidogyne incognita* and growth of tomato. Biocontrol Sci. Technol..

[184] Peçen A.K., Galip M.I. (2013). Nematicidal efficacies of several organic and microbial fertilizers against Root-knot nematodes (*Meloidogyne* spp.) in organic tomato farming. Turk. Entomoloji Derg..

[185] Hashem M., Abo-Elyousr K.A. (2011). Management of the root-knot nematode *Meloidogyne incognita* on tomato with combinations of different biocontrol organisms. Crop Prot..

[186] Oclarit E., Cumagun C. (2009). Evaluation of efficacy of *Paecilomyces lilacinus* as biological control agent of *Meloidogyne incognita* attacking tomato. J. Plant Prot. Res..

[187] Kaşkavalcı G., Tuzel Y., Dura O., Oztekin G.B. (2009). Effects of Alternative Control Methods Against *Meloidogyne incognita* in Organic Tomato Production. Ekoloji.

[188] Goswami B.K., Mittal A. (2004). Management of root-knot nematode infecting tomato by *Trichoderma viride* and *Paecilomyces lilacinus*. Indian Phytopathol..

[189] Parajuli G., Kemerait R., Timper P. (2014). Improving suppression of *Meloidogyne spp*. by *Purpureocillium lilacinum* strain 251. Nematology.

[190] Xiang N., Lawrence K.S., Donald P.A. (2018). Biological control potential of plant growth-promoting rhizobacteria suppression of *Meloidogyne incognita* on cotton and *Heterodera glycines* on soybean: A review. J. Phytopathol..

[191] Huang W.K., Cui J.K., Liu S.M., Kong L.A., Wu Q.S., Peng H. (2016). Testing various biocontrol agents against the root-knot nematode (*Meloidogyne incognita*) in cucumber plants identifies a combination of *Syncephalastrum racemosum* and *Paecilomyces lilacinus* as being most effective. Biol. Control..

[192] Sharma A., Sharma S., Yadav S., Naik S.N. (2014). Role of Karanja deoiled cake based medium in production of protease and fatty acids by *Paecilomyces lilacinus* 6029. J. Biosci. Bioeng..

[193] Teixeira H., Monteiro A.C., Vilela A.W. (2010). Uso de agentes microbianos e químico para o controle de *Meloidogyne incognita* em soja. Acta Sci. Agron..

[194] Sharma P., Pandey R. (2009). Biological control of root-knot nematode; *Meloidogyne incognita* in the medicinal plant; *Withania somnifera* and the effect of biocontrol agents on plant growth. Afr. J. Agric. Res..

[195] Bontempo A.F., Fernandes R.H., Lopes J., Freitas L.G., Lopes E.A. (2014). *Pochonia chlamydosporia* controls *Meloidogyne incognita* on carrot. Australas. Plant Pathol..

[196] Bhat M.Y., Wani A.H., Fazal M. (2012). Effect of *Paecilomyces lilacinus* and plant growth promoting rhizobacteria on *Meloidogyne incognita* inoculated black gram, Vigna mungo plants. J. Biopest..

[197] Peiris P.U.S., Li Y., Brown P., Xu C. (2020). Fungal biocontrol against *Meloidogyne spp*. in agricultural crops: A systematic review and meta-analysis. Biol. Control..

[198] Kaşkavalcı G., Hatice A. (2012). Efficacy of the combined usage of several control methods against Root-knot nematodes (*Meloidogyne spp.*) in organic tomato agriculture. Turk. Entomoloji Derg..

[199] Siddiqui I.A., Qureshi S.A., Sultana V., Ehteshamul-Haque S., Ghaffar A. (2000). Biological control of root rot-root knot disease complex of tomato. Plant Soil.

[200] Mokbel A.A., Alharbi A.A. (2014). Suppressive effect of some microbial agents on root-knot nematode, *Meloidogyne javanica* infected eggplant *Aus*. J. Crop Sci..

[201] Abo-Korah M.S. (2017). Biological control of root-knot nematode, *Meloidogyne javanica* infecting ground cherry, using two nematophagous and mychorrhizal Fungi. Egypt. J. Biol. Pest Control.

[202] Sun M.H., Gao L., Shi Y.X., Li B.J., Liu X.Z. (2006). Fungi and actinomycetes associated with *Meloidogyne* spp. eggs and females in China and their biocontrol potential. J. Invertebr. Pathol..

[203] Bonants P.J.M., Fitters P.F.L., Thijs H., Den Belder E., Waalwijk C., Henfling J.W.D.M. (1995). A basic serine protease from *Paecilomyces lilacinus* with biological activity against *Meloidogyne hapla* eggs. Microbiology.

[204] Kiewnick S., Lueth P., Sikora R.A. (2002). Development of a biocontrol product based on *Paecilomyces lilacinus* (strain 251). Phytopathology.

[205] Souza E.C., Coelho L., Lemes E.M., Gontijo L.N. (2019). Manejo de *Meloidogyne exigua* em seringueira com produtos biológicos e químicos. Summa Phytopathol..

[206] Akram S., Khan S., Javed N., Ahmad S. (2020). Integrated Management of Root Knot Nematode *Meloidogyne graminicola* Golden and Birchfield Parasitizing on Wheat. Pak. J. Zool..

[207] Starr J.L., Ong K.L., Huddleston M., Handoo Z.A. (2007). Controle of *Meloidogyne marylandi* on Bermudagrass. Nematropica.

[208] Cadioli M.C., Santiago D.C., Hoshino A.T., Homechin M. (2007). Crescimento micelial e parasitismo de *Paecilomyces lilacinus* sobre ovos de *Meloidogyne paranaensis* em diferentes temperaturas “in vitro”. Ciência Agrotecnologia.

[209] Santiago D.C., Homechin M., Silva J.F.V., Ribeiro E.R., Gomes B.C., Santoro P.H. (2006). Seleção de isolados de *Paecilomyces lilacinus* (Thom.) Samson para controle de *Meloidogyne paranaensis* em tomateiro. Cienc. Rural..

[210] Zhu M.L., Mo M.H., Xia Z.Y., Li Y.H., Yang S.J., Li T.F., Zhan K.Q. (2006). Detection of two fungal biocontrol agents against root-knot nematodes by RAPD markers. Mycopathologia.

[211] Khan M.R., Altaf S., Mohidin F.A., Khan U., Anwer A. (2009). Biological control of plant nematodes with phosphate- solubilizing microorganisms. Phosphate Solubilizing Microbes Crop Improv..

[212] Westphal A., Becker J.O. (2001). Impact of soil suppressiveness on various population densities of *Heterodera schachtii*. Ann. Appl. Biol..

[213] Olivares-Bernabeu C.M., López-Llorca L.V. (2002). Fungal egg-parasites of plant-parasitic nematodes from Spanish soils. Rev. Iberoam. Micol..

[214] Chen S.Y., Dickson D.W., Mitchell D.J. (1996). Pathogenicity of fungi to eggs of *Heterodera glycines*. J. Nematol..

[215] Hay F.S., Skipp R.A. (1993). Fungi and Actinomycetes Associated with Cysts of Heterodera Trifolii Goffart (Nematoda: Tylenchida) In Pasture Soils in New Zealand. Nematologica.

[216] Bernard E.C., Self L.H., Tyler D.D. (1997). Fungal parasitism of soybean cyst nematode, *Heterodera glycines* (Nemata: Heteroderidae), in differing cropping-tillage regimes. Appl. Soil Ecol..

[217] Kepenekçi İ., Toktay H., Oksal E., Buzboğa R., İmren M. (2018). Effect of *Purpureocillium lilacinum* on root lesion nematode, *Pratylenchus thornei*. J. Agric. Sci..

[218] Misterlaine M.K.R., Chaves A., Dilma D.A., Da Silva E.J., Walber W.D. (2011). Controle biológico de fitonematóides do gênero *Pratylenchus* a través de inoculante natural em cana-de- açúcar. Rev. Bras. Cienc. Agrar..

[219] Botha-Greeff M.S., Van Biljon E.R. (1999). Integrated Nematode Control on Cotton in South Africa: Present status. CORESTA Meet. Agron. Phyt..

[220] Castillo J.D., Lawrence K.S., Kloepper J.W., Van Santen E. (2010). Evaluation of *Drechslerella dactyloides*, *Drechslerella brochopaga*, and *Paecilomyces lilacinus* for the biocontrol of *Rotylenchulus reniformis*. Nematropica.

[221] Gené J., Verdejo-Lucas S., Stchigel A.M., Sorribas F.J., Guarro J. (2005). Microbial parasites associated with *Tylenchulus semipenetrans* in citrus orchards of Catalonia, Spain. Biocontrol Sci. Technol..

[222] Hammam M.M.A., Wafaa M.E.N., Abd-Elgawad M.M.M. (2016). Biological and chemical control of the citrus nematode, *Tylenchulus semipenetrans* (Cobb, 1913) on mandarin in Egypt. Egypt. J. Biol. Pest Control.

[223] Esnard J., Marban-mendoza N., Zuckerman B.M. (1998). Effects of three microbial broth cultures and an organic amendment on growth and populations of free living and plant-parasitic nematodes on banana. Eur. J. Plant Pathol..

[224] López-Lima D., Sánchez-Nava P., Carrión G., Núñez-Sánchez A.E. (2013). 89 % Reduction of a potato cyst nematode population using biological control and rotation. Agron. Sustain. Dev..

[225] Faria M., Wraight S.P. (2001). Biological control of *Bemisia tabaci* with fungi. Crop Prot..

[226] Sanjaya Y. (2016). Pathogenicity of three entomopathogenic fungi, *Metarhizium anisopliae*, *Beauveria bassiana*, and *Paecilomyces lilacinus*, to *Tetranychus kanzawai* infesting papaya seedlings. Arthropods.

[227] Jackson M.A., Cliquet S., Iten L.B. (2003). Media and fermentation processes for the rapid production of high concentrations of stable blastospores of the bioinsecticidal fungus *Paecilomyces fumosoroseus*. Biocontrol Sci. Technol..

[228] Jackson M.A., Erhan S., Poprawski T.J. (2006). Influence of formulation additives on the desiccation tolerance and storage stability of blastospores of the entomopathogenic fungus *Paecilomyces fumosoroseus* (Deuteromycotina: *Hyphomycetes*). Biocontrol Sci. Technol..

[229] Ruiu L. (2018). Microbial biopesticides in agroecosystems. Agronomy.

[230] Beris E.I., Papachristos D.P., Fytrou A., Antonatos S.A., Kontodimas D.C. (2013). Pathogenicity of three entomopathogenic fungi on pupae and adults of the Mediterranean fruit fly, *Ceratitis capitata* (Diptera: Tephritidae). J. Pest Sci..

[231] Panyasiri C., Attathom T., Poehling H.M. (2007). Pathogenicity of entomopathogenic fungi-potential candidates to control insect pests on tomato under protected cultivation in Thailand. J. Plant Dis. Prot..

[232] Hoy M.A., Singh R., Rogers M.E. (2010). Evaluations of a novel isolate of *Isaria fumosorosea* for control of the Asian citrus psyllid, *Diaphorina citri* (Hemiptera: *Psyllidae*). Fla. Entomol..

[233] Yeo H., Pell J.K., Alderson P.G., Clark S.J., Pye B.J. (2003). Laboratory evaluation of temperature effects on the germination and growth of entomopathogenic fungi and on their pathogenicity to two aphid species. Pest Manag. Sci..

[234] Del Prado E.N., Iannacone J., Gómez H. (2008). Effect of two entomopathogenic fungi in controlling *Aleurodicus cocois* (Curtis, 1846) (Hemiptera: Aleyrodidae). Chil. J. Agric. Res..

[235] Hussein H.M., Skoková O., Půža V., Zemek R. (2016). Laboratory evaluation of *Isaria fumosorosea* CCM 8367 and *Steinernema feltiae* Ustinov against immature stages of the Colorado potato beetle. PLoS ONE.

[236] Hunter W.B., Avery P.B., Pick D., Powell C.A. (2011). Broad spectrum potential of *Isaria fumosorosea* against insect pests of citrus. Fla. Entomol..

[237] Lekimme M., Focant C., Farnir F., Mignon B., Losson B. (2008). Pathogenicity and thermotolerance of entomopathogenic fungi for the control of the scab mite, *Psoroptes ovis*. Exp. Appl. Acarol..

[238] Kang B.R., Han J.H., Kim J.J., Kim Y.C. (2018). Dual biocontrol potential of the entomopathogenic fungus, *Isaria javanica*, for both aphids and plant fungal pathogens. Mycobiology.

[239] Dunlap C.A., Jackson M.A., Wright M.S. (2007). A foam formulation of *Paecilomyces fumosoroseus*, an entomopathogenic biocontrol agent. Biocontrol Sci. Technol..

[240] El-Sharabasy H.M. (2015). Laboratory evaluation of the effect of the entomopathogenic fungi, *Hirsutella thompsonii* and *Paecilomyces fumosoroseus*, against the citrus brown mite, *Eutetranychus orientalis* (Acari: Tetranychidae) *Plant Prot*. Sci..

[241] Jessica J.J., Peng T.L., Sajap A.S., Lee S.H., Syazwan S.A. (2019). Evaluation of the virulence of entomopathogenic fungus, *Isaria fumosorosea* isolates against subterranean termites *Coptotermes spp.* (Isoptera: Rhinotermitidae). J. For. Res..

[242] Ansari M.A., Brownbridge M., Shah F.A., Butt T.M. (2008). Efficacy of entomopathogenic fungi against soil-dwelling life stages of western flower thrips, *Frankliniella occidentalis*, in plant-growing media. Entomol. Exp. Appl..

[243] Fiedler Ż., Sosnowska D. (2007). Nematophagous fungus *Paecilomyces lilacinus* (Thom) Samson is also a biological agent for control of greenhouse insects and mite pests. BioControl.

[244] Luangsa-Ard J.J., Berkaew P., Ridkaew R., Hywel-Jones N.L., Isaka M. (2009). A beauvericin hot spot in the genus *Isaria*. Mycol. Res..

[245] Lee Y.S., Han J.H., Kang B.R., Kim Y.C. (2019). Dibutyl succinate, produced by an insect-pathogenic fungus, *Isaria javanica* pf185, is a metabolite that controls of aphids and a fungal disease, anthracnose. Pest Manag. Sci..

[246] Xie L., Han J.H., Kim J.J., Lee S.Y. (2016). Effects of culture conditions on conidial production of the sweet potato whitefly pathogenic fungus *Isaria javanica*. Mycoscience.

[247] Ishii M., Takeshita J., Ishiyama M., Tani M., Koike M., Aiuchi D. (2015). Evaluation of the pathogenicity and infectivity of entomopathogenic hypocrealean fungi, isolated from wild mosquitoes in Japan and Burkina Faso, against female adult *Anopheles stephensi* mosquitoes. Fungal Ecol..

[248] Goffré D., Folgarait P.J. (2015). *Purpureocillium lilacinum*, potential agent for biological control of the leaf-cutting ant *Acromyrmex lundii*. J. Invertebr. Pathol..

[249] Bakeri S.A., Ali S.R.A., Tajuddin N.S., Kamaruzzaman N.E. (2009). Efficacy of entomopathogenic fungi, *Paecilomyces spp*., In controlling the oil palm bag worm, *Pteroma pendula* (Joannis). J. Oil Palm Res..

[250] Saito T., Takatsuka J., Shimazu M. (2012). Characterization of *Paecilomyces cinnamomeus* from the camellia whitefly, *Aleurocanthus camelliae* (Hemiptera: Aleyrodidae), infesting tea in Japan. J. Invertebr. Pathol..

[251] Dal Bello G., Padin S., Lopez Lastra C., Fabrizio M. (2001). Laboratory evaluation of chemical-biological control of the rice weevil (*Sitophilus oryzae* L.) in stored grains. J. Stored Prod. Res..

[252] Usanmaz-Bozhuyuk A., Kordali S., Keddek M., Simsek D., Altinok M.A., Altinok H.H., Komaki A. (2018). Mortality efeects of six different entomopathogenic funfi strains on rice weevil, *Sitophilus oryzae* (L.) (Coleoptera: Curculionidae). Fresenuis Environ. Bull..

[253] Lefort F., Fleury D., Fleury I., Coutant C., Kuske S., Kehrli P., Maignet P. (2014). Pathogenicity of entomopathogenic fungi to the green peach aphid *Myzus persicae* sulzer (Aphididae) and the european tarnished bug *Lygus rugulipennis* poppius (Miridae). Egypt. J. Biol. Pest Control.

[254] Demirci F., Muştu M., Kaydan M.B., Ülgentürk S. (2011). Laboratory evaluation of the effectiveness of the entomopathogen; *Isaria farinosa*, on citrus mealybug, *Planococcus Citri*. J. Pest. Sci..

[255] Komaki A., Kordali Ş., Bozhüyük A.U., Altinok H.H., Kesdek M., Şimşek D., Altinok M.A. (2017). Laboratory assessment for biological control of *Tribolium confusum* du Val., 1863 (Coleoptera: Tenebrionidae) by entomopathogenic fungi. Turk. Entomoloji Derg..

[256] Führer E., Rosner S., Schmied A., Wegensteiner R. (2001). Studies on the significance of pathogenic fungi in the population dynamics of the lesser spruce sawfly, *Pristiphora abietina* christ. (Hym., Tenthredinidae). J. Appl. Entomol..

[257] Davidson G., Chandler D. (2005). Laboratory evaluation of entomopathogenic fungi against larvae and adults of *Onion maggot* (Diptera: Anthomyiidae). J. Econ. Entomol..

[258] Parker B.L., Skinner M., Costa S.D., Gouli S., Reid W., El Bouhssini M. (2003). Entomopathogenic fungi of *Eurygaster integriceps* Puton (Hemiptera: Scutelleridae): Collection and characterization for development. Biol. Control..

[259] Vega F.E., Mercadier G., Damon A., Kirk A. (1999). Natural enemies of the coffee berry borer, *Hypothenemus hampei* (Ferrari) (Coleoptera: Scolytidae) in Togo and Cote d’lvoire, and other insects associated with coffee beans. Afr. Entomol..

[260] Rose E.A.F., Harris R.J., Glare T.R., Rose E.A.F. (1999). Possible pathogens of social wasps (hymenoptera: Vespidae) and their potential as biological control agents. N. Zeal. J. Zool..

[261] Oliveira I., Pereira J.A., Lino-Neto T., Bento A., Baptista P. (2015). Plant-mediated effects on entomopathogenic fungi: How the olive tree influences fungal enemies of the olive moth, *Prays oleae*. BioControl.

[262] Rodriguez-Rueda D., Fargues J. (1980). Pathogenicity of entomopathogenic hyphomycetes, *Paecilomyces fumosoroseus* and *Nomuraea rileyi*, to eggs of noctuids, *Mamestra brassicae* and *Spodoptera littoralis*. J. Invertebr. Pathol..

[263] Ansari M.A., Vestergaard S., Tirry L., Moens M. (2004). Selection of a highly virulent fungal isolate, *Metarhizium anisopliae* CLO 53, for controlling *Hoplia philanthus*. J. Invertebr. Pathol..

[264] Shapiro-Ilan D.I., Cottrell T.E., Jackson M.A., Wood B.W. (2008). Virulence of Hypocreales fungi to pecan aphids (Hemiptera: Aphididae) in the laboratory. J. Invertebr. Pathol..

[265] Vandenberg J.D., Jackson M.A., Lacey L.A. (1998). Relative Efficacy of Blastospores and Aerial Conidia of *Paecilomyces fumosoroseus* against the Russian Wheat Aphid. Invertebr. Pathol..

[266] Altre J.A., Vandenberg J.D., Cantone F.A. (2003). Pathogenicity of *Paecilomyces fumosoroseus* isolates to diamondback moth, *Plutella xylostella*: Correlation with spore size, germination speed, and attachment to cuticle. Biocontrol. Sci. Technol..

[267] Ansari M.A., Evans M., Butt T.M. (2009). Identification of pathogenic strains of entomopathogenic nematodes and fungi for wireworm control. Crop Prot..

[268] Castillo M.A., Moya P., Hernández E., Primo-Yúfera E. (2000). Susceptibility of *Ceratitis capitata* Wiedemann (Diptera: Tephritidae) to entomopathogenic fungi and their extracts. Biol. Control.

[269] Hesketh H., Alderson P.G., Pye B.J., Pell J.K. (2008). The development and multiple uses of a standardised bioassay method to select hypocrealean fungi for biological control of aphids. Biol. Control.

[270] Vidal C., Osborne L.S., Lacey L.A., Fargues J. (1998). Effect of host plant on the potential of *Paecilomyces fumosoroseus* (Deuteromycotina: Hyphomycetes) for controlling the silverleaf whitefly, *Bemisia argentifolii* (Homoptera: Aleyrodidae) in greenhouses. Biol. Control..

[271] Subandiyah S., Nikoh N., Sato H., Wagiman F., Tsuyumu S., Fukatsu S. (2000). Isolation and characterization of two entomopathogenic fungi attacking *Diaphorina citri* (Homoptera, Psylloidea) in Indonesia. Mycoscience.

[272] Castineiras A., Peña J.E., Duncan R., Osborne L. (1996). Potential of *Beauveria bassiana* and *Paecilomyces fumosoroseus* (Deuteromycotina: Hyphomycetes) as Biological Control Agents of *Thrips palmi* (Thysanoptera: Thripidae). Fla. Entomol..

[273] Lezama-Gutiérrez R., Hamm J.J., Molina-ochoa J., López-Edwards M., Pescador-Rubio A., Gonzalez-Ramirez M., Styer E.L. (2001). Occurrence of Entomopathogens of *Spodoptera frugiperda* (Lepidoptera: Noctuidae) in the Mexican States of Michoacán, Colima, Jalisco and Tamaulipas *Fla*. Entomol..

[274] Alma C.R., Goettel M.S., Roitberg B.D., Gillespie D.R. (2007). Combined effects of the entomopathogenic fungus, *Paecilomyces fumosoroseus* Apopka-97, and the generalist predator, *Dicyphus hesperus*, on whitefly populations. BioControl.

[275] Gökçe A., Er M.K. (2005). Pathogenicity of *Paecilomyces spp.* to the glasshouse whitefly, *Trialeurodes vaporariorum*, with some observations on the fungal infection process. Turk. J. Agric. For..

[276] Chan-Cupul W., Ruiz-Sánchez E., Cristóbal-Alejo J., Pérez-Gutiérrez A., Munguía-Rosales R., Lara-Reyna J. (2010). Desarrollo in vitro de cuatro cepas nativas de *Paecilomyces fumosoroseus* y su patogenicidad en estados inmaduros de mosquita blanca. Agrociencia.

[277] Afifi A.M., Ali F.S., El-Saiedy E.M.A., Ahmed M.M. (2015). Compatibility and integration of some control methods for controlling *Tetranychus urticae* Koch infesting tomato plants in Egypt. Egypt. J. Biol. Pest Control.

[278] Poprawski T.J., Parker P.E., Tsai J.H. (1999). Laboratory and field evaluation of hyphomycete insect pathogenic fungi for control of brown citrus aphid (Homoptera: Aphididae). Environ. Entomol..

[279] Vasilev P., Andreev R., Palagacheva N., Kutinkova H., Stefanova D. (2019). Efficacy of non-chemical insecticides against *Hyalopterus pruni* (Hemiptera: Aphididae) on plum. J. Biopest..

[280] Roy H.E., Cottrell T.E. (2008). Forgotten natural enemies: Interactions between coccinellids and insect-parasitic fungi. Eur. J. Entomol..

[281] Ganassi S., Moretti A., Stornelli C., Fratello B., Pagliai A.M.B., Logrieco A., Sabatini M.A. (2001). Effect of *Fusarium*, *Paecilomyces* and *Trichoderma* formulations against aphid *Schizaphis graminum*. Mycopathologia.

[282] Akey D.H., Hennebery T.J. (1998). Control of silverleaf whitefly with the entomopathogenic fungi, *Paecilomyces fumosoroseus* and *Beauveria bassiana* in upland cotton in Arizona. Proc. Beltwide Cott. Conf..

[283] Clifton E.H., Jaronski S.T., Hajek A.E. (2020). Virulence of Commercialized Fungal Entomopathogens Against Asian Longhorned Beetle (Coleoptera: Cerambycidae). J. Insect Sci..

[284] Vandenberg J.D., Sandvol L.E., Jaronski S.T., Jackson M.A., Souza E.J., Halbert S.E. (2001). Efficacy of fungi for control of Russian wheat aphid (Homoptera: Aphididae) in irrigated wheat. Southwest Entomol..

[285] Vänninen I., Hokkanen H., Tyni-Juslin J. (1999). Attempts to control cabbage root flies *Delia radicum* L. and *Delia floralis* (Fall,) (Dipt., Anthomyiidae) with entomopathogenic fungi: Laboratory and greenhouse tests. J. Appl. Entomol..

[286] Sookar P., Bhagwant S., Ouna E.A. (2008). Isolation of entomopathogenic fungi from the soil and their pathogenicity to two fruit fly species (Diptera: Tephritidae). J. Appl. Entomol..

[287] Daniel C., Wyss E. (2009). Susceptibility of different life stages of the European cherry fruit fly, *Rhagoletis cerasi*, to entomopathogenic fungi. J. Appl. Entomol..

[288] Sahagún C.A.A., Gutiérrez R.L., Ochoa J.M., Velasco E.G., Edwards M.L., Domínguez O.R., Vázquez C.C., Velázquez W.P.R., Skoda S.R., Foster J.E. (2005). Susceptibility of biological stages of the horn fly, *Haematobia irritans*, to entomopathogenic fungi (Hyphomycetes). J. Insect Sci..

[289] Zemek R., Hussein H.M., Prenerová E. (2012). Laboratory evaluation of *Isaria fumosorosea* against *Spodoptera littoralis*. Commun. Agric. Appl. Biol. Sci..

[290] Behle R.W., Gutierrez C.G., Guerra P.T., McGuire M.R., Jackson M.A. (2006). Pathogenicity of blastospores and conidia of *Paecilomyces fumosoroseus* against larvae of the Mexican bean beetle, *Epilachna varivestis* mulsant. Southwest Entomol..

[291] Peña J.E., Osborne L.S., Duncan R.E. (1996). Potential of Fungi As Biocontrol Agents of *Polyphagotarsonemus latus* (Acari: Tarsonemidae). Entomophaga.

[292] Poprawski T.J., Jones W.J. (2001). Host plant effects on activity of the mitosporic fungi *Beauveria bassiana* and *Paecilomyces fumosoroseus* against two populations of *Bemisia* whiteflies (Homoptera: Aleyrodidae). Mycopathologia.

[293] Lacey L.A., Kirk A.A., Millar L., Mercadier G., Vidal C. (1999). Ovicidal and larvicidal activity of conidia and blastospores of *Paecilomyces fumosoroseus* (Deuteromycotina: Hyphomycetes) against *Bemisia argentifolii* (Homoptera: Aleyrodidae) with a description of a bioassay system allowing prolonged survival of control. Biocontrol Sci. Technol..

[294] Dong T., Zhang B., Jiang Y., Hu Q. (2016). Isolation and classification of fungal whitefly entomopathogens from soils of Qinghai-Tibet Plateau and Gansu Corridor in China. PLoS ONE.

[295] James R.R. (2003). Combining Azadirachtin and *Paecilomyces fumosoroseus* (Deuteromycotina: Hyphomycetes) to Control *Bemisia argentifolii* (Homoptera: Aleyrodidae). J. Econ. Entomol..

[296] Wraight S.P., Carruthers R.I., Bradley C.A., Jaronski S.T., Lacey L.A., Wood P., Galani-Wraight S. (1998). Pathogenicity of the Entomopathogenic Fungi *Paecilomyces spp.* And *Beauveria bassiana* against the Silverleaf Whitefly,*Bemisia argentifolii*. J. Invertebr. Pathol..

[297] Nian X.G., He Y.R., Lu L.H., Zhao R. (2015). Evaluation of the time-concentration-mortality responses of *Plutella xylostella* larvae to the interaction of *Isaria fumosorosea* with the insecticides beta-cypermethrin and *Bacillus thuringiensis*. Pest Manag. Sci..

[298] Wang X., Xu J., Wang X., Qiu B., Cuthbertson A.G.S., Du C., Wu J., Ali S. (2019). *Isaria fumosorosea*-based zero-valent iron nanoparticles affect the growth and survival of sweet potato whitefly, *Bemisia tabaci* (Gennadius). Pest Manag. Sci..

[299] Scorsetti A.C., Humber R.A., De Gregorio C., López Lastra C.C. (2008). New records of entomopathogenic fungi infecting *Bemisia tabaci* and *Trialeurodes vaporariorum*, pests of horticultural crops, in Argentina. BioControl.

[300] Poprawski T.J., Legaspi J.C., Parker P.E. (1998). Influence of Entomopathogenic Fungi on *Serangium parcesetosum* (Coleoptera: Coccinellidae), an Important Predator of Whiteflies (Homoptera: Aleyrodidae). Environ. Entomol..

[301] Woltz J.M., Donahue K.M., Bruck D.J., Lee J.C. (2015). Efficacy of commercially available predators, nematodes and fungal entomopathogens for augmentative control of *Drosophila suzukii*. J. Appl. Entomol..

[302] Scholz-Döbelin V.P., Stockmann S., Rheinland L., Bonn P., Ösnabrück F.H. (2003). Mycoinsecticides against Whitefly *Trialeurodes vaporariorum* in Tomatoe. Gesunde Pflanz..

[303] Öztürk H.E., Güven Ö., Karaca I. (2015). Effects of Some Bioinsecticides and Entomopathogenic Fungi on Colorado Potato Beetle (Leptinotarsa Decemlineata L.). Commun. Agric. Appl. Biol. Sci..

[304] Lohmeyer K.H., Miller J.A. (2006). Pathogenicity of three formulations of entomopathogenic fungi for control of adult *Haematobia irritans* (Diptera: Muscidae). J. Econ. Entomol..

[305] Leles R.N., Sousa N.A., Rocha L.F.N., Santos A.H., Silva H.H.G., Luz C. (2010). Pathogenicity of some hypocrealean fungi to adult *Aedes aegypti* (Diptera: Culicidae). Parasitol. Res..

[306] Kepenekci I., Oksal E., Saglam H.D., Atay T., Tulek A., Evlice E. (2015). Identification of Turkish isolate of the entomopathogenic fungi, *Purpureocillium lilacinum* (syn: Paecilomyces lilacinus) and its effect on potato pests, *Phthorimaea operculella* (Zeller) (Lepidoptera: Gelechiidae) and *Leptinotarsa decemlineata* (Say) (Coleoptera; Chrysomelidae). Egypt. J. Biol. Pest Control.

[307] Medeiros F.R., Nonata R., De Lemos S., Alice A., Rodrigues C., Filho A.B., Oliveira L.J.M.G., Araújo J.R.G. (2017). Occurrence of *Purpureocillium lilacinum* in Citrus Black Fly Nymphs. Rev. Bras. Frutic..

[308] Amatuzzi R.F., Cardoso N., Poltronieri A.S., Poitevin C.G., Dalzoto P., Zawadeneak M.A., Pimentel L.C. (2018). Potential of endophytic fungi as biocontrol agents of *Duponchelia fovealis* (Zeller) (lepidoptera:Crambidae). Braz. J. Biol..

[309] Angelo I.C., Fernandes É.K.K., Bahiense T.C., Perinotto W.M.S., Golo P.S., Moraes A.P.R., Biitencourt V.R.E.P. (2012). Virulence of *Isaria sp*. and *Purpureocillium lilacinum* to *Rhipicephalus microplus* tick under laboratory conditions. Parasitol. Res..

[310] Demirci F., Denizhan E. (2010). Paecilomyces lilacinus, a potential biocontrol agent on apple rust mite Aculus schlechtendali and interactions with some fungicides in vitro. Phytoparasitica.

[311] Debnath S., Sreerama Kumar P. (2017). Fungi associated with mortality of the red spider mite, *Oligonychus coffeae* nietner (Acari: Tetranychidae), a serious pest of tea in North-Eastern India. Egypt. J. Biol. Pest. Control.

[312] Imoulan A., Alaoui A., Meziane A.E. (2011). Natural occurrence of soil-borne entomopathogenic fungi in the Moroccan Endemic forest of *Argania spinosa* and their pathogenicity to *Ceratitis capitata*. World J. Microbiol. Biotechnol..

[313] Baydar R., Güven Ö., Karaca I. (2016). Occurrence of entomopathogenic fungi in agricultural soils from isparta province in turkey and their pathogenicity to *Galleria mellonella* (L.) (lepidoptera: Pyralidae) larvae. Egypt. J. Biol. Pest Control.

[314] Woolfolk S., Stokes C.E., Watson C., Baker G., Brown R., Baird R. (2016). Fungi associated with *Solenopsis invicta* buren (Red Imported Fire Ant, Hymenoptera: Formicidae) from mounds in Mississippi. Southeast Nat..

[315] Meng X., Hu J., Ouyang G. (2017). The isolation and identification of pathogenic fungi from *Tessaratoma papillosa* Drury (Hemiptera: Tessaratomidae). PeerJ.

[316] Ahmed B.I. (2010). Potentials of entomopathogenic fungi in controlling the menace of maize weevil *Sitophilus zeamais* Motsch (Coleoptera: Curculinidae) on stored maize grain. Arch. Phytopathol. Plant Prot..

[317] Berón C.M., Diaz B.M. (2005). Pathogenicity of hyphomycetous fungi against *Cyclocephala signaticollis*. BioControl.

[318] Saruhan I., Erper I., Tuncer C., Uçak H., Öksel C., Akça I. (2014). Evaluation of some commercial products of entomopathogenic fungi as biocontrol agents for *Aphis fabae* scopoli (Hemiptera: Aphididae). Egypt. J. Biol. Pest Control.

[319] Zawadneak M.A.C., Pimentel I.C., Robl D., Dalzoto P., Vicente V., Sosa-Gómez D.R., Porsani M., Cuquel F.M. (2015). Registro de *Paecilomyces niveus* Stolk & Samson, 1971 (Ascomycota: Thermoascaceae) como patógeno de *Nasonovia ribisnigri* (Mosley, 1841) (Hemiptera, Aphididae) no Brasil. Braz. J. Biol..

[320] Vega-Aquino P., Sanchez-Peña S., Blanco C.A. (2010). Activity of oil-formulated conidia of the fungal entomopathogens *Nomuraea rileyi* and *Isaria tenuipes* against lepidopterous larvae. J. Invertebr. Pathol..

[321] Bruck D.J. (2004). Natural occurrence of entomopathogens in pacific northwest nursery soils and their virulence to the black vine weevil, *Otiorhynchus sulcatus* (F.) (Coleoptera: Curculionidae). Environ. Entomol..

[322] Baksh A., Khan A. (2012). Pathogenicity of *Paecilomyces tenuipes* to diamond back moth, *Plutella xylostella* at three temperatures in Trinidad. Int. J. Agric. Biol..

[323] Moorthi P.V., Balasubramanian C., Ramar M., Murugan K. (2015). Biocontrol Potential of Entomopathogenic Fungi against *Spodoptera Litura*. Sci. Agric..

[324] Fahmy B.F.G., Ghadir N.M.F.A., Manaa S.H., Abou Ghadir M.F. (2015). Occurrence of entomopathogenic fungi in grain aphids in upper egypt, with reference to certain pathogenic tests using scanning electron microscope. Egypt. J. Biol. Pest Control.

[325] Abd-ElAzeem E.M., El-Medany W.A.Z., Sabry H.M. (2019). Biological activities of spores and metabolites of some fungal isolates on certain aspects of the spiny bollworms *Earias insulana* (Boisd.) (Lepidoptera: Noctuidae). Egypt. J. Biol. Pest Control.

[326] Liu H., Skinner M., Parker B.L., Brownbridge M. (2002). Pathogenicity of *Beauveria bassiana*, *Metarhizium anisopliae* (Deuteromycotina: Hyphomycetes), and other entomopathogenic fungi against *Lygus lineolaris* (Hemiptera: Miridae). J. Econ. Entomol..

[327] Medina W.F., Sulvarán J.A.R., Rieche A.K.S. (2013). Efecto de las cepas nativas *Paecilomyces* sp. (bainier) y *lecanicillium* sp. (zimm) en el control de *Carmenta foraseminis* Eichlin (lepidoptera: Sesiidae) en cultivos de cacao (theobroma cacao l.). Acta Agron..

[328] Jaramillo J., Borgemeister C. (2006). New bioassay method to assess the pathogenicity of Colombian strains of *Metarhizium anisopliae* (Metsch.) Sorokin and *Paecilomyces sp*. (Deuteromycotina: Hyphomycetes) against the subterranean burrower bug Cyrtomenus bergi Froeschner (Hemiptera: Cydnidae). J. Invertebr. Pathol..

[329] Hou F.J., Addis S.N.K., Azmi W.A. (2018). Virulence evaluation of entomopathogenic fungi against the red palm weevil, *Rhynchophorus ferrugineus* (Coleoptera: Dryopthoridae). Malays. Appl. Biol..

[330] Cabanillas H.E., Jones W.A. (2009). Effects of temperature and culture media on vegetative growth of an entomopathogenic fungus *Isaria sp*. (Hypocreales: Clavicipitaceae) naturally affecting the whitefly, *bemisia tabaci* in Texas. Mycopathologia.

[331] Zulfitri A., Lestari A.S., Krishanti N.P.R.A., Zulfiana D. (2018). Laboratory Evaluation of the Selected Entomopathogenic Fungi and Bacteria. Against Larval and Pupal Stages of *Spodoptera litura* L.. IOP. Conf. Ser. Earth Environ. Sci..

[332] Leite M.S.P., Iede E.T., Penteado S.R.C., Zaleski S.R.M., Camargo J.M.M., Ribeiro R.D. (2011). Seleção de isolados de fungos entomopatogênicos para o controle de *Hedypathes betulinus* e avaliação da persistência. Floresta.

